# High-pathogenicity island enhances NDM-5 activity and drives global spread of *Escherichia coli* ST410

**DOI:** 10.1093/ismejo/wrag086

**Published:** 2026-04-11

**Authors:** Wanyun He, Jieying Tu, Zhongpeng Cai, Zixuan Wang, Chuying Liang, Yingbo Gao, Guolong Gao, Luchao Lv, Jun Yang, Yi-Yun Liu, Jianzhong Shen, Yang Wang, Jian-Hua Liu

**Affiliations:** State Key Laboratory for Animal Disease Control and Prevention, Key Laboratory of Zoonosis of Ministry of Agricultural and Rural Affairs, College of Veterinary Medicine, South China Agricultural University, Guangzhou, Guangdong 510642, China; State Key Laboratory for Animal Disease Control and Prevention, Key Laboratory of Zoonosis of Ministry of Agricultural and Rural Affairs, College of Veterinary Medicine, South China Agricultural University, Guangzhou, Guangdong 510642, China; State Key Laboratory for Animal Disease Control and Prevention, Key Laboratory of Zoonosis of Ministry of Agricultural and Rural Affairs, College of Veterinary Medicine, South China Agricultural University, Guangzhou, Guangdong 510642, China; State Key Laboratory for Animal Disease Control and Prevention, Key Laboratory of Zoonosis of Ministry of Agricultural and Rural Affairs, College of Veterinary Medicine, South China Agricultural University, Guangzhou, Guangdong 510642, China; State Key Laboratory for Animal Disease Control and Prevention, Key Laboratory of Zoonosis of Ministry of Agricultural and Rural Affairs, College of Veterinary Medicine, South China Agricultural University, Guangzhou, Guangdong 510642, China; State Key Laboratory for Animal Disease Control and Prevention, Key Laboratory of Zoonosis of Ministry of Agricultural and Rural Affairs, College of Veterinary Medicine, South China Agricultural University, Guangzhou, Guangdong 510642, China; State Key Laboratory for Animal Disease Control and Prevention, Key Laboratory of Zoonosis of Ministry of Agricultural and Rural Affairs, College of Veterinary Medicine, South China Agricultural University, Guangzhou, Guangdong 510642, China; State Key Laboratory for Animal Disease Control and Prevention, Key Laboratory of Zoonosis of Ministry of Agricultural and Rural Affairs, College of Veterinary Medicine, South China Agricultural University, Guangzhou, Guangdong 510642, China; Guangdong Provincial Key Laboratory of Pharmaceutical Bioactive Substances, School of Basic Medical Sciences, Guangdong Pharmaceutical University, Guangzhou, Guangdong 510006, China; State Key Laboratory for Animal Disease Control and Prevention, Key Laboratory of Zoonosis of Ministry of Agricultural and Rural Affairs, College of Veterinary Medicine, South China Agricultural University, Guangzhou, Guangdong 510642, China; ational Key Laboratory of Veterinary Public Health Security, College of Veterinary Medicine, China Agricultural University, Beijing 100193, China; National Key Laboratory of Veterinary Public Health Security, College of Veterinary Medicine, China Agricultural University, Beijing 100193, China; State Key Laboratory for Animal Disease Control and Prevention, Key Laboratory of Zoonosis of Ministry of Agricultural and Rural Affairs, College of Veterinary Medicine, South China Agricultural University, Guangzhou, Guangdong 510642, China

**Keywords:** *Escherichia coli* ST410, ecological adaptation, metal acquisition, resistance–virulence co-evolution, high-risk clones

## Abstract

The international high-risk clone *Escherichia coli* ST410 poses a serious threat to clinical therapy and public health. By analyzing 3434 ST410 genomes, we observed a broad ecological and geographic distribution along with frequent acquisition of the carbapenem resistance gene *bla*_NDM-5_ and a yersiniabactin-encoding high-pathogenicity island (HPI). We found that *bla*_NDM-5_ and HPI co-occurred in the most successful subclades. Functional assays comparing the wild-type strain with its isogenic mutant demonstrated that HPI augments bacterial iron uptake, promotes growth under iron-limited conditions, and increases pathogenicity in a mouse infection model. HPI also increases intracellular zinc levels and boosts NDM-5 β-lactamase activity, thereby improving bacterial survival under imipenem exposure *in vitro* and in mice. These findings suggest that the successful dissemination of ST410 is propelled by a synergy between HPI-mediated metal acquisition and *bla*_NDM-5_, linking enhanced virulence with elevated antibiotic resistance. This study provides mechanistic insight into resistance–virulence co-evolution in an emerging bacterial lineage and illustrates how ecological selection imposed by metal limitation and antibiotic exposure can jointly shape the success of high-risk clones across diverse ecosystems.

## Introduction

Antimicrobial resistance has become a pressing global health crisis, with projections of up to 40 million deaths annually by 2050 if effective interventions are not implemented [1]. In response, the 2024 World Health Organization (WHO) list of critical-priority pathogens includes carbapenem-resistant *Enterobacterales*, underscoring the urgency for enhanced surveillance and new control strategies against these pathogens [2]. Among carbapenem-resistant *Enterobacterales*, *E. coli* stands out due to its adaptability and pandemic potential. Recent epidemiological studies indicate a shift in dominant carbapenem-resistant *E. coli* (CREC) lineages—from highly virulent ST131 and ST38 to newer, highly drug-resistant clones such as ST410 and ST167 [3]. It has been observed that *E. coli* ST410 has rapidly emerged as a globally disseminated clone. Initially predominant in Europe and North America [4–9], ST410 has since expanded across Asia and other continents in both clinical and environmental settings [10–15]. Beyond hospital settings, *E. coli* ST410 has been documented across interconnected ecological compartments, including wastewater systems, food chains, and animal reservoirs [16–19]. This broad ecological distribution underscores the relevance of a “One Health” perspective (an integrated, unifying approach that aims to sustainably balance and optimize the health of people, animals, and ecosystems) and suggests that ST410 is exposed to heterogeneous selective pressures across host-associated and environmental niches, which may collectively shape its population structure, resistance repertoire, and evolutionary trajectory.

Phylogenomic studies have revealed that ST410 has undergone a stepwise evolutionary trajectory [20–22]. Early diversification gave rise to two major clades defined by distinct *fimH* alleles: the A/H53 clade (*fimH53*) and the B1/H24 clade (*fimH24*). Within the B lineage, subsequent subclades show a progressive accumulation of antimicrobial resistance determinants, beginning with chromosomal mutations in quinolone resistance-determining region (QRDR) (B2/H24R), followed by the acquisition of the extended-spectrum beta-lactamase (ESBL) gene *bla*_CTX-M-15_ on a multi-replicon IncF plasmid (F36:31:A4:B1) (B3/H24Rx), and later the incorporation of carbapenemase genes, including *bla*_OXA-181_ on an IncX3 plasmid and *bla*_NDM-5_ on a *bla*_CTX-M-15_-carrying IncF plasmid (F1:A1:B49) (B4/H24RxC). More recently, the B5/H24RxC subclone has been recognized as an emerging lineage associated with outbreaks in pediatric and companion animal hospitals in China and has also been detected in multiple geographic regions worldwide [23, 24]. This subclone appears to have evolved from the B4/H24RxC background through a series of genetic events, retaining the *bla*_NDM-5_-positive multi-replicon IncF plasmid from its ancestor but losing the *bla*_OXA-181_-bearing IncX3 plasmid, and uniquely acquiring a novel O-antigen (Onovel1) and a yersiniabactin-encoding high-pathogenicity island (HPI), a feature presumed to enhance virulence [23]. Despite these advances in understanding the evolutionary history and global dissemination of ST410, the ecological and functional consequences of its key adaptive traits remain poorly understood.

A growing body of evidence suggests that certain bacterial lineages can overcome the typical trade-off between antimicrobial resistance and virulence by acquiring compensatory genetic factors. For instance, the globally disseminated carbapenem-resistant *Klebsiella pneumoniae* ST11 has achieved a “high resistance–high virulence” phenotype through acquisition of a pLVPK-like virulence plasmid [25, 26]. By analogy, we suspect the success of *E. coli* ST410 may involve a similar convergence of resistance and virulence traits. The recent emergence of the B5/H24RxC subclone, which carries the HPI in addition to *bla*_NDM-5_, provides a strong hint of such a convergence. The HPI is a 102-kb chromosomal genomic island flanked by IS*100* elements that was originally identified in highly pathogenic *Yersinia* species, where it mediates yersiniabactin-dependent iron acquisition. Following its initial characterization, this locus was formally designated as a “high-pathogenicity island” (HPI) [27, 28]. Subsequent studies have demonstrated that HPI is found in diverse members of the *Enterobacterales*, including *E. coli*, *Klebsiella*, *Enterobacter*, and *Citrobacter* [29–31]. In *E. coli*, the HPI typically spans 35–45 kb, which usually lacks flanking insertion sequences and only encodes the yersiniabactin siderophore system, organized into four operons: (i) *fyuA*, (ii) *irp2*-*irp1*-*ybtU*-*ybtT*-*ybtE*, (iii) *ybtA*, and (iv) *ybtP*-*ybtQ*-*ybtX*-*ybtS* [32]. This system enhances iron acquisition and is a known virulence factor in extraintestinal pathogenic *E. coli* (ExPEC) [33, 34]. However, it has not been experimentally or population-genomically demonstrated whether HPI specifically contributes to the pandemic success of ST410, nor whether it provides a measurable selective advantage in this lineage. Moreover, although the presence of HPI in B5/H24RxC has been noted [23], its distribution and potential impact across other ST410 subclones have not been explored.

In this study, we address these knowledge gaps by integrating comparative genomics with targeted assays. We aimed to (i) delineate the distribution of HPI and other genetic characterizations across the global ST410 population and (ii) determine how HPI influences key phenotypes such as metal acquisition, antibiotic resistance, and virulence. Through this approach, we uncover a synergistic interaction between a virulence island and a carbapenemase gene that underlies the success of ST410, providing novel insights into how resistance and virulence co-evolve in an emerging bacterial lineage.

## Materials and methods

### Bacterial genome collection

Whole-genome assemblies of *Enterobacterales* were retrieved from the GenBank database (accessed on 31 December 2024). Assemblies corresponding to *E. coli* genomes were extracted and subjected to *in silico* multi-locus sequence typing (MLST) using MLST v2.23.0 [35] to identify ST410 isolates. Duplicate assemblies deposited in GenBank were removed, retaining only the most recent submission to ensure data uniqueness. Assembly quality was evaluated using QUAST v5.3.0 [36], and only assemblies with N50 > 50 000, total genome length between 4.5 and 5.5 Mb, and <200 contigs were retained. Assembly completeness was further assessed using BUSCO v6.0.0 [37], and only assemblies with ≥95% completeness were included for downstream analyses. In addition, whole-genome assemblies of 97 *E. coli* ST410 strains that were isolated, sequenced, and previously published by our laboratory were included in this study [24, 38–41]. Metadata associated with each genome, including year of collection, isolation source, and geographic origin, were retrieved from BioSample records and are summarized in Supplementary Data 1.

### Phylogenetic and population structure analysis

Pairwise average nucleotide identity (ANI) between ST410 genomes was calculated using FastANI v1.34, a *k*-mer-based method that enables rapid estimation of genomic similarity among closely related isolates. To further resolve their evolutionary relationships, a core genome alignment was generated using snippy-core from Snippy v4.6.0 (https://github.com/tseemann/snippy), with a complete ST410 chromosome (GenBank accession no. CP104851.1) as the reference genome. This alignment, which includes both single-nucleotide polymorphisms (SNPs) and invariant sites, was subsequently cleaned to remove ambiguous and non-standard nucleotide characters by snippy-clean_full_aln function. To minimize the impact of homologous recombination on phylogenetic inference, recombination regions were identified and filtered using Gubbins v3.4.3 [42], which applies iterative phylogenetic reconstruction using RAxML. The recombination-filtered core genome alignment was used to infer a maximum-likelihood phylogeny with IQ-TREE v3.0.1 [43]. The optimal substitution model (TVM) was selected automatically by ModelFinder, and branch support was assessed with 1000 ultrafast bootstrap replicates. The final phylogenetic tree was visualized and annotated by the iTOL web server [44].

To delineate the fine-scale population structure within ST410, we extracted SNPs from the same recombination-filtered alignment using SNP-sites v2.5.1 [45]. These SNP data served as input for hierarchical Bayesian clustering via rhierbaps package v1.1.4 in R v4.5.2 [46, 47] and for pairwise SNP distance calculation using snp-dists v0.8.2 (https://github.com/AdmiralenOla/snp-dists).

### Genetic characterization

FimH alleles were identified using ABRicate v1.0.1 (https://github.com/tseemann/abricate) against a curated *fimH* allele database (https://bitbucket.org/genomicepidemiology/fimtyper_db). O- and H-antigen types were determined using SerotypeFinder v2.0 [48]. For isolates with untypable O-antigens, *wcaM* and *hisI* genes flanking the O-antigen gene cluster were used as boundaries to extract the complete O-antigen gene cluster. Extracted sequences were compared and visualized using Easyfig v2.1 [49]. QRDR mutations were identified using PointFinder v4.1.1 [50]. Antimicrobial resistance genes, plasmid replicon types, and virulence factors were detected using ABRicate v1.0.1 against the ResFinder [51], PlasmidFinder [52], and VFDB [53] databases, respectively. Plasmid multilocus sequence typing (pMLST) was performed for IncF plasmids using the pMLST scheme implemented in PlasmidFinder and the PubMLST database (https://pubmlst.org/plasmid/). The presence and distribution of HPI were assessed by screening for genes involved in yersiniabactin synthesis (*irp2*, *irp1*, *ybtU*, *ybtT*, *ybtE*, *ybtS*), transport (*ybtP*, *ybtQ*, *ybtX*), receptor (*fyuA*), and regulation (*ybtA*).

For large-scale screening across all *E. coli* genomes available in GenBank, a custom reference library was constructed using the complete HPI sequence from ST410 (CP104851.1). In parallel, a curated *bla*_NDM_ allele library derived from the ResFinder database (https://bitbucket.org/genomicepidemiology/resfinder_db/src/master/) was used to screen for *bla*_NDM_ gene. Screening was performed separately for HPI-positive and HPI-negative genomes to assess co-occurrence patterns.

### RNA isolation and reverse transcription-quantitative PCR

To investigate the transcriptional regulation of HPI genes, reverse transcription-quantitative PCR (RT-qPCR) analysis was performed on ST410 strains (GYX208DH6-1, BioProject no. PRJNA842647; AHM7C101I, PRJNA842647), which were previously isolated from a food market environment and a chicken farm environment, respectively [39, 40]. Total RNA was extracted from exponential-phase bacterial cultures grown in Luria–Bertani (LB) broth using the E.Z.N.A. Bacterial RNA Kit (Omega Bio-tek) according to the manufacturer’s protocol. Briefly, bacterial cells were harvested by centrifugation (3000 × g) and subjected to cell wall digestion. Cell lysis was achieved using the lysis buffer, followed by vigorous vortexing and centrifugation (13 000 × g) to obtain clarified lysates. The resulting lysate was mixed with ethanol and applied to silica membrane columns for RNA purification. Following sequential washing steps to remove contaminants, high-quality RNA was eluted with nuclease-free water and immediately stored at −80°C.

For gene expression analysis, complementary DNA (cDNA) synthesis was performed using HiScript III All-in-one RT SuperMix (Vazyme) following the manufacturer’s instructions. Quantitative PCR amplification was performed using ChamQ Universal SYBR qPCR Master Mix (Vazyme) with specific primer pairs for HPI genes. The 16S ribosomal RNA (rRNA) was employed as an endogenous reference gene. Relative gene expression levels were calculated using the 2^-∆∆CT^ method [54].

The bacterial strains and plasmids used in this study are described in Supplementary Table 1, and all primers employed are provided in Supplementary Table 2.

### Gene knockout

Gene knockouts were performed using a dual-plasmid CRISPR-Cas9 system comprising pCasKP and pSGKP vectors [55]. The pCasKP plasmid was introduced into *E. coli* strain AHM7C101I [designated wild type (WT)] via electroporation and selected on LB agar plates containing 30 μg/ml chloromycin. Successful transformation was confirmed by PCR amplification using primers check-cas-F/check-cas-R, and Cas9 expression was subsequently induced with 0.2% arabinose.

Two distinct pSGKP targeting plasmids were constructed for specific gene knockouts. The *irp2*-targeted pSGKP plasmid was assembled using primer pairs RE-sg-F/irp2-sg-R and irp2-sg-F/RE-sg-R, and the *bla*_NDM-5_-targeted pSGKP plasmid was constructed using primer pairs RE-sg-F/NDM5-sg-R and NDM5-sg-F/RE-sg-R. All PCR products were purified using the E.Z.N.A. Cycle Pure Kit (Omega Bio-tek) and assembled using the Gib-XKL Fast Seamless Cloning Kit (Xinkailai Biotechnology). Ligation products were transformed into *E. coli* DH5α competent cells and selected on LB agar plates containing 50 μg/ml rifampicin. Successful recombinant plasmids were verified by PCR amplification and Sanger sequencing using primers check-sg-F/check-sg-R. In addition, a repair template for the *irp2* knockout was constructed using primer pairs irp2-F1/irp2-R1 and irp2-F2/irp2-R2 to facilitate homology-directed repair.

For the *irp2* knockout, both the targeting pSGKP plasmid and the repair template were co-electroporated into the AHM7C101I/pCasKP strain to enable precise gene replacement via homologous recombination. In contrast, for the elimination of the *bla*_NDM-5_ gene located on the IncX3 plasmid of the AHM7C101I strain, only the targeting pSGKP plasmid was used. Successful gene knockouts were confirmed by PCR amplification and Sanger sequencing using primers check-irp2-F/check-irp2-R and check-NDM5-F/check-NDM5-R, respectively. Helper plasmids (pCasKP and pSGKP) were subsequently cured by overnight culture in LB medium containing 5% sucrose at 37°C. The resulting knockout strains were designated WT∆*irp2* and WT∆*bla*_NDM-5_.

### Intracellular metal measurements by inductively coupled plasma optical emission spectrometry

To determine the impact of *irp2* gene deletion on metal acquisition capacity, the intracellular metal content of WT and WT∆*irp2* strains was quantified using intracellular metal measurements by inductively coupled plasma optical emission spectrometry (ICP-OES). Overnight bacterial cultures grown in MH broth were subcultured at a 1% inoculum into 300 ml of fresh medium and cultivated to the exponential phase at 37°C. Cells were harvested by centrifugation at 3000 × g, followed by extensive washing with sterile ddH_2_O to remove extracellular metal contaminants and culture medium residues that could interfere with accurate intracellular metal measurements.

The washed cell pellets were resuspended and subjected to mechanical disruption using a high-pressure homogenizer at 900 bar and maintained at 4°C to preserve metal–protein complexes and prevent oxidative degradation. The total protein concentration in the resulting lysates was determined using the bicinchoninic acid (BCA) assay (Epizyme Biotech) to normalize metal content per unit of cellular protein. The clarified lysates were subsequently subjected to acid digestion until complete solubilization was achieved, as evidenced by the formation of clear, particle-free solutions suitable for spectroscopic analysis. Multi-element quantification was performed by ICP-OES (iCAP 7000, Thermo Fisher Scientific) using established standard metal detection parameters.

### Bacterial growth curve

To evaluate the functional impact of *irp2* gene deletion on bacterial growth fitness under iron-depleted conditions, growth curves of WT and WT∆*irp2* strains were assessed. Exponential-phase bacterial suspensions were inoculated into LB medium containing either 200 μM 2,2′-dipyridyl (DIP) or 1 mg/ml human transferrin (hTf), to simulate iron-limited environments. Growth was monitored using 96-well microplates, with each well containing 199 μl of medium and 1 μl of standardized bacterial suspension to ensure consistent initial inoculum density. Blank controls contained medium only. The microplates were incubated at 37°C in a microplate reader (Ensight, PerkinElmer) with optical density measurements at 600 nm (OD_600_) recorded at 30-min intervals over 12 h to generate growth curves.

To evaluate the functional impact of *irp2* gene deletion on survival ability under imipenem pressure, growth curves of WT and WT∆*irp2* strains in MH medium containing 8 μg/ml imipenem (IPM) were assessed.

### NDM-5 β-lactamase production and purification

The *bla*_NDM-5_ gene of the AHM7C101I strain was cloned into the pET28a expression vector using seamless cloning technology, with primer pairs pET28-F/pET28-R and NDM5-ET28-F/NDM5-ET28-R. The resulting recombinant plasmid was transformed into *E. coli* BL21(DE3) competent cells and selected on LB agar plates containing 50 μg/ml kanamycin. Successful cloning was verified by PCR amplification and Sanger sequencing using primers check-pET28-F/check-pET28-R.

For protein production, the verified recombinant strain was cultured in LB medium and induced with 500 μM isopropyl β-D-1-thiogalactopyranoside (IPTG) at 16°C for 16 h. Following induction, bacterial cells were harvested by centrifugation and subjected to mechanical lysis by a high-pressure homogenizer (900 bar, 4°C). The His-tagged NDM-5 protein was purified from clarified lysates using nickel affinity chromatography with stepwise imidazole gradient elution ranging from 0 to 500 μM. Purified fractions were analyzed by sodium dodecyl sulfate–polyacrylamide gel electrophoresis (SDS-PAGE) to assess purity and molecular weight consistency. The purified NDM-5 protein exhibited a molecular weight >25 kDa and was successfully eluted with 200 μM imidazole during purification. To ensure long-term stability and remove residual imidazole, the purified protein was extensively dialyzed against phosphate-buffered saline (PBS) containing 10% glycerol as a cryoprotectant. Final protein concentrations were determined using the BCA assay, and aliquots were stored at −80°C.

### Checkerboard assay

A two-dimensional concentration 8 × 8 matrix was established in sterile 96-well microtiter plates to evaluate ethylenediaminetetraacetic acid (EDTA)–imipenem interactions. EDTA was subjected to two-fold serial dilutions along the vertical axis (50 μl per well; 100 μM to 0 μM), and imipenem was diluted two-fold along the horizontal axis (50 μl per well; 16 μg/ml to 0 μg/ml or 0.5 μg/ml to 0 μg/ml). Well H1 served as the positive growth control. Further, 100 μl of diluted bacterial suspension was added to each well. Plates were incubated statically at 37°C overnight, and bacterial growth was quantified by measuring OD_600_ using a microplate reader (Multiskan Spectrum, Thermo Fisher Scientific).

### Evaluation of bacterial virulence and imipenem susceptibility in mice

To evaluate the functional impact of the *irp2* gene deletion on bacterial virulence, comprehensive *in vivo* infection studies were conducted using a murine sepsis model. WT and WT∆*irp2* strains were cultured to the exponential phase, harvested, washed with sterile physiological saline, and resuspended in sterile saline to standardized concentrations for consistent inoculum delivery. SPF-grade six-week-old female C57BL/6 mice were purchased (ZhuHai BesTest Bio-Tech) and received a seven-day acclimatization.

For bacterial pathogenicity assessment, survival curve experiments were performed using two experimental groups (*n* = 10/group) that received intraperitoneal infections with 100 μl of bacterial suspension (2.5 × 10^7^ colony-forming units, CFUs), alongside an uninfected control group (*n* = 8). Infected mice were monitored every 12 h for 120 h postinfection. For bacterial burden analysis, mice (two groups, *n* = 10/group) were infected intraperitoneally with 100 μl bacterial suspension (1 × 10^8^ CFUs) and humanely euthanized at 12 h postinfection. Organs, including liver, spleen, lung, and kidney, were aseptically collected, weighed for standardization, mechanically homogenized, and serially diluted for CFU enumeration on LB agar plates containing 0.5 μg/ml meropenem. To directly compare the competitive fitness of WT versus WT∆*irp2* strains within the host environment, co-infection competition assays were performed using mice (*n* = 8) that received mixed inocula containing equal proportions of both strains (2 × 10^7^ CFUs each) via intraperitoneal injection. Blood samples were collected from the tail vein and serially diluted for CFU enumeration on LB agar plates containing 0.5 μg/ml meropenem. WT and WTΔ*irp2* strains were differentiated by PCR amplification using primer pairs irp2-F1/irp2-R2.

For imipenem susceptibility evaluation under clinically relevant conditions, therapeutic efficacy studies were conducted using mice infected intraperitoneally with 2.5 × 10^7^ CFUs of either strain (two groups, *n* = 8/group), followed by imipenem/cilastatin (IPM/CIL) treatment (50 mg/kg) administered 12 h postinfection to simulate delayed therapeutic intervention scenarios commonly encountered in clinical settings. Additionally, competitive fitness under imipenem pressure was assessed using co-infected mice (*n* = 8) that received mixed bacterial inocula (4 × 10^7^ CFUs each), followed by imipenem/cilastatin treatment (50 mg/kg) at 12 h postinfection. Bacterial load in circulating blood was quantified through the tail vein sampling, followed by serial dilution plating on selective media. WT and WTΔ*irp2* strains were differentiated by PCR amplification using primer pairs irp2-F1/irp2-R2.

### Ethic approval

All experimental procedures involving laboratory animals were conducted in strict accordance with institutional guidelines and approved by the Experimental Animal Ethics Committee of South China Agricultural University (Ethics approval no. 2024c028 and 2025c127).

### Data visualization

Plots were generated using R v4.5.2 and GraphPad Prism v10.2.0. Figures were assembled and annotated using Adobe Illustrator v2021. World maps were obtained from the Standard Map Service System of China (http://bzdt.ch.mnr.gov.cn/) in compliance with national cartographic standards [approval no. GS(2016)2968]. Illustrative elements (including map icons and schematic symbols) were sourced from Freepik (https://www.freepik.com/) under the Freepik Standard License.

### Statistical analysis

Statistical analyses were performed using GraphPad Prism v10.2.0. Associations between categorical variables were evaluated using the chi-square test. For group comparisons (WT versus WTΔ*irp2*), data distributions were assessed for normality, and homogeneity of variances was tested before analysis. Survival data from mouse infection experiments were analyzed using Kaplan–Meier curves, with differences assessed by the Log-rank test. Statistical significance is indicated as *P* < .05 (^*^), *P* < .01 (^**^), *P* < .001 (^***^), and *P* < .0001 (^****^).

## Results

### Global footprint of ST410 with recent clonal sweeps in B3/H24Rx_2 and B5/H24RxC

To reconstruct the phylogeny of ST410, we assembled 3434 genomes, including 3337 publicly available strains from the GenBank database and 97 strains from our laboratory collection (Supplementary Data 1). Hierarchical Bayesian clustering at level 1 grouped these isolates into five major clades (Supplementary Data 2), which corresponded closely to previously defined ST410 subclones. Specifically, Clade 2 comprised the A/H53 subclone (*n* = 55), Clade 3 corresponded to B1/H24 (*n* = 151), Clade 4 aligned with B2/H24R (*n* = 541), and Clade 1 matched B3/H24Rx (*n* = 1336). Both B4/H24RxC (*n* = 737) and B5/H24RxC (*n* = 614) were grouped within Clade 5 at level 1 but were clearly distinguished by level 2 clustering. In addition, level 2 clustering further subdivided B3/H24Rx into two distinct lineages, designated B3/H24Rx_1 (*n* = 529) and B3/H24Rx_2 (*n* = 807).

**Figure 1:**
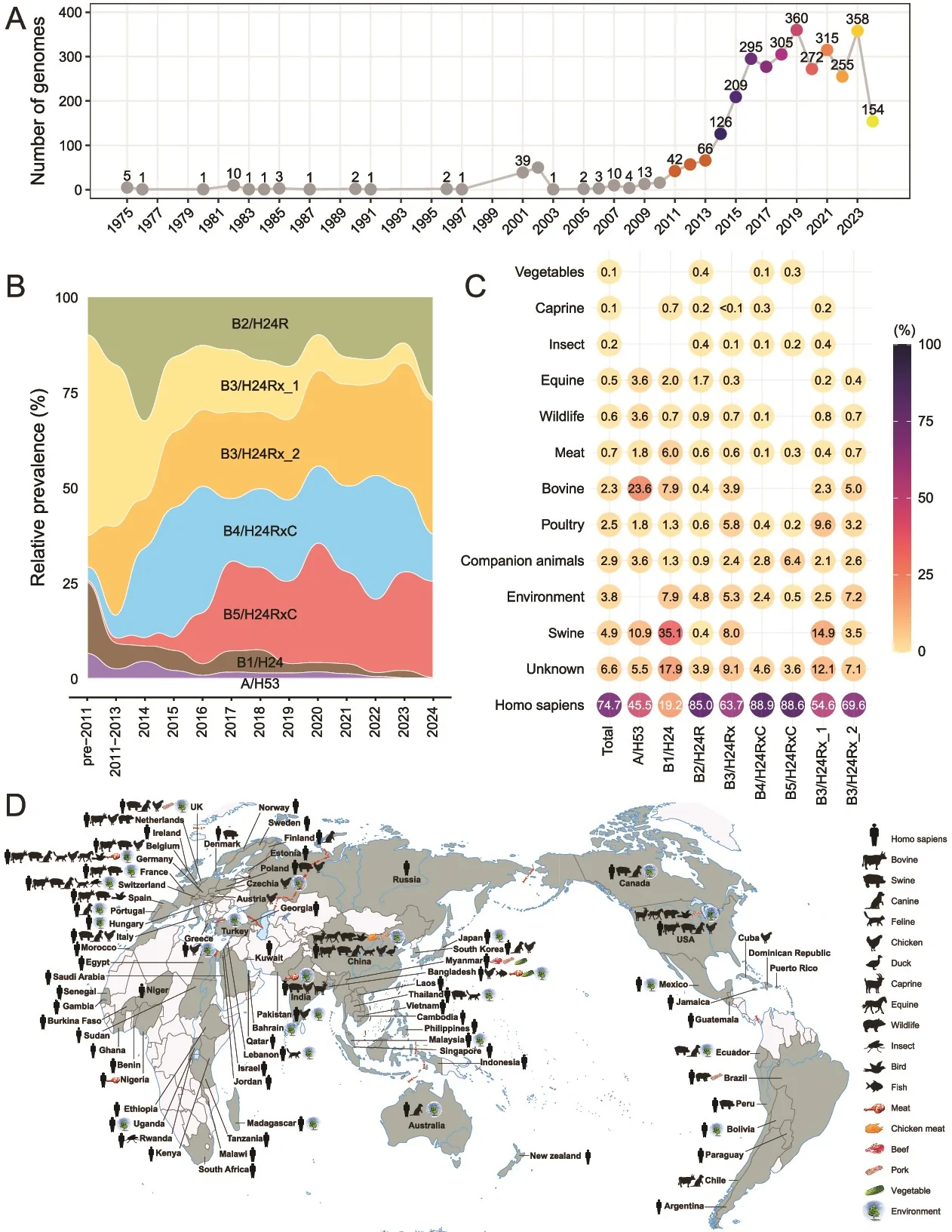
Epidemiological distribution of *E. coli* ST410. (A) Temporal distribution showing the number of ST410 genomes collected per year, with counts shown above each data point. (B) Temporal dynamics of major ST410 subclones, showing changes in their relative abundance over time based on collection date metadata. Percentage represents the proportion of each subclone among ST410 isolates within a given time period. (C) Ecological distribution of ST410 subclones across different isolation sources. Circle size reflects the relative abundance (%) of each subclone within a given ecological category, as derived from available metadata. (D) Global geographical distribution of ST410 isolates. Countries from which ST410 isolates have been identified are shaded in gray. Symbols indicate isolation sources, as annotated in the legend, and are placed at approximate geographic locations for visualization purposes. All distributions are descriptive of publicly available metadata and should be interpreted cautiously, given heterogeneous sampling.

Temporal analysis revealed an increase in the number of ST410 genomes over time, particularly after 2013, reflecting the rapid expansion of genome sequencing and improved global surveillance (Fig. 1A). Based on available collection dates, the A/H53 and B1/H24 subclones comprised the earliest ST410 isolates, traceable to as far back as 1975, followed by a gradual decline in relative representation over time (Fig. 1B; Supplementary Data 1). The A/H53 subclone has not been detected since 2023. In contrast, B2/H24R, B3/H24Rx, and B4/H24RxC subclones were first observed in 2001, 2001, and 2007, respectively, whereas the B5/H24RxC subclone emerged more recently, first appearing in 2015 and increasing in relative frequency thereafter. Within the B3/H24Rx group, there was a clear succession: B3/H24Rx_1 predominated in the mid-2000s but declined during the 2010s, whereas B3/H24Rx_2 (first seen in 2007) steadily rose to prominence. Together, these temporal patterns suggest ongoing clonal replacement events within ST410, with more recent subclades (B3/H24Rx_2 and B5/H24RxC) expanding at the expense of older ones.

We examined the ecological sources of ST410 isolates using available metadata. ST410 was found in a wide variety of hosts and environments, underscoring its ecological versatility. Humans represented the predominant reservoir (2564/3434), but ST410 was also recovered from food-producing animals (swine, *n* = 168; bovine, *n* = 79; poultry, *n* = 87), companion animals (*n* = 101), wildlife (*n* = 19), food products (meat, *n* = 24; vegetables, *n* = 5), and environmental samples (*n* = 130) (Fig. 1C), reflecting the ability of ST410 to persist, adapt, and evolve across interconnected ecological niches. Different subclones exhibited distinct host association patterns. For example, the B2/H24R subclone had the broadest host range, followed by B3/H24Rx. Within B3/H24Rx, B3/H24Rx_2 was relatively restricted to humans compared to B3/H24Rx_1, although a minority of isolates originated from food animals and environmental sources. In contrast, the hyper-successful B5/H24RxC subclone showed a striking preference for human hosts (544/614) with a minor portion from companion animals (*n* = 39), suggesting this lineage may be particularly adapted to human-associated environments and perhaps capable of human-to-human transmission, with pets as potential vectors.

Global mapping of ST410 revealed its presence in 80 countries spanning six continents (Fig. 1D). However, the geographic spread of individual subclones varied (Supplementary Figs 1–7). The early lineage A/H53 was detected in only 12 countries, mostly in Europe and the USA, possibly reflecting its historical confinement or limited sampling outside those regions. B1/H24 had a moderately broad distribution (23 countries), with concentrations in Europe and America. B2/H24R showed a substantial global footprint (41 countries across Europe, Asia, Africa, and America). B3/H24Rx was the most widespread, found in 66 countries across six continents; within this group, B3/H24Rx_2 achieved the wider reach (54 countries) than B3/H24Rx_1 (45 countries). B4/H24RxC was detected in 43 countries, with major hotspots in China, India, and Bangladesh. By contrast, the B5/H24RxC subclone—despite its virulence and resistance profile—had the most limited geographical range (20 countries), found predominantly in Asia, which may be attributed to its recent emergence.

To assess genomic similarity among lineages, we calculated ANI values. The ANI values between A/H53 and B/H24 isolates were generally below 99.5%; however, no substantial ANI differences were observed among the five B/H24 subclones, with most pairwise comparisons exceeding 99.5% (Supplementary Data 2). These results not only confirm the high genomic similarity within B/H24 subclones but also suggest the limited resolution of ANI for distinguishing closely related lineages at this scale. To further resolve fine-scale genetic differences, we performed pairwise core genome SNP distance analysis. This revealed that isolates within B5/H24RxC subclone had the lowest genetic diversity, with distances ≤27 SNPs (median, 9 SNPs), compared with substantially higher median distances observed in other subclones (Supplementary Fig. 8 and Supplementary Data 2). Similarly, within the B3/H24Rx subclone, B3/H24Rx_2 showed a narrower SNP range (median, 23 SNPs) than its earlier counterparts, B3/H24Rx_1 (median, 29 SNPs). These patterns indicate that both B5/H24RxC and B3/H24Rx_2 have undergone rapid clonal expansion with limited diversification, a genomic signature typical of successful emerging lineages that have experienced recent selective sweeps, likely due to the acquisition of advantageous traits.

In summary, ST410 has demonstrated a remarkable capacity to colonize diverse niches and to spread internationally. A/H53 and B1/H24 represent earlier, geographically restricted branches. The recently emerged B5/H24RxC subclone, though not yet the most geographically widespread, exhibits limited genetic diversity and a strong preference for human hosts, suggesting they are in the early stages of a clonal expansion driven by newly acquired adaptive traits. The newly differentiated B3/H24Rx_2 subclade within B3/H24Rx displays extensive ecological and geographic distribution, also suggesting its successful expansion following the acquisition of adaptive traits.

### Stepwise resistance escalation, antigenic shift, and HPI–NDM-5 convergence define ST410 evolution

**Figure 2:**
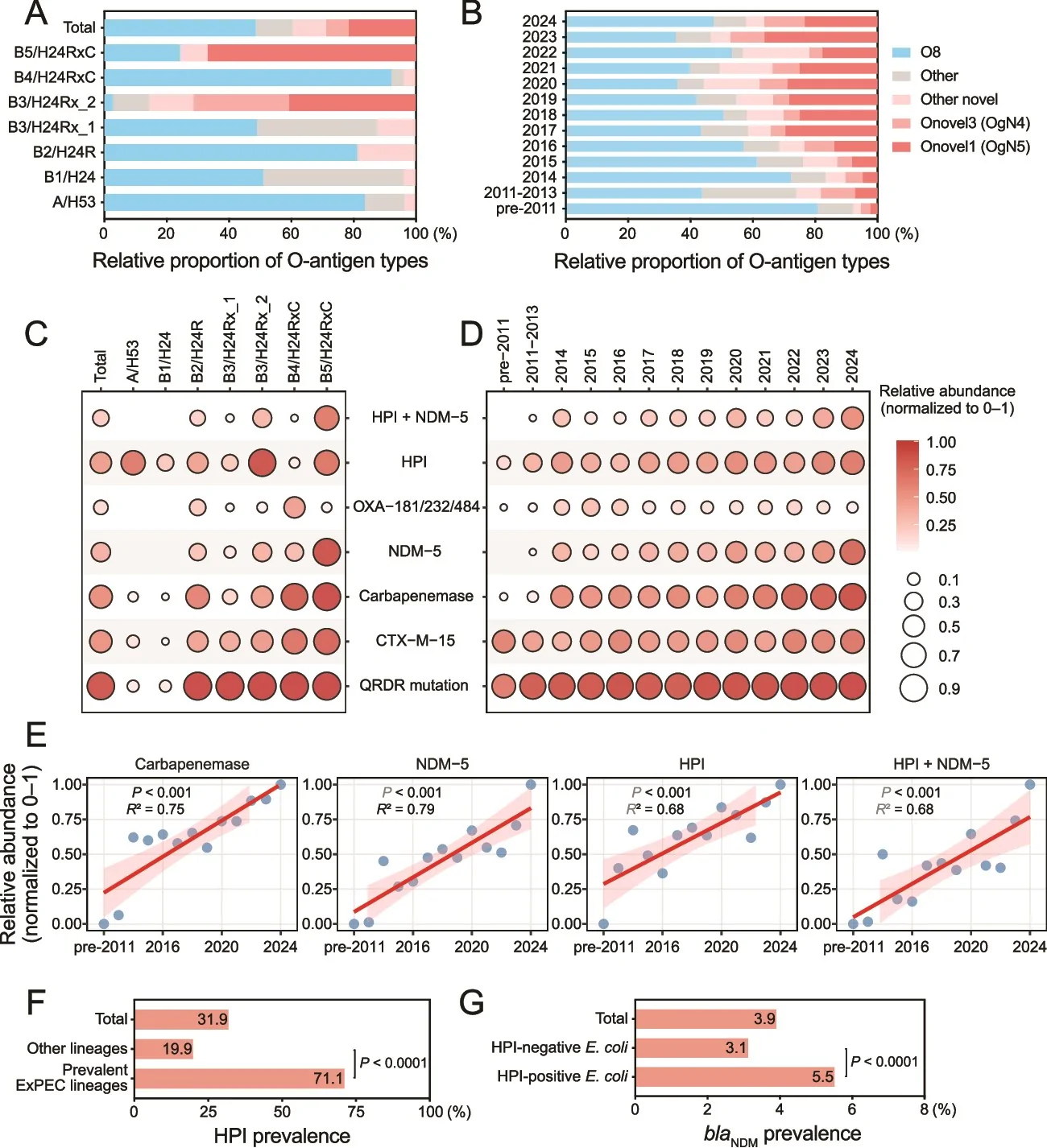
Genetic characteristics and evolutionary trends of *E. coli* ST410. (A) Relative abundance of O-antigen types across major ST410 subclones. Relative abundance represents the proportion of isolates carrying a given O-antigen within each subclone. (B) Temporal changes in the relative abundance of O-antigen types within ST410, stratified by year of collection. (C) Relative abundance of key resistance and virulence determinants among ST410 subclones, normalized to a 0–1 scale. (D) Relative abundance of resistance and virulence determinants across different time periods, normalized to a 0–1 scale. (E) Temporal trends in the relative abundance of resistance and virulence determinants in ST410 over time (normalized to 0–1). Shaded areas indicate 95% confidence intervals. Pearson correlation analysis with linear regression was applied to assess temporal associations. The coefficient of determination (*R*^2^) was calculated using linear models. Statistical significance was evaluated using two-sided Pearson correlation tests. (F) Prevalence of the HPI among *E. coli* genomes retrieved from the GenBank database, shown for representative ExPEC lineages. (G) Prevalence of *bla*_NDM_ among *E. coli* genomes retrieved from the GenBank database, stratified by HPI-positive and HPI-negative isolates. All temporal patterns shown are descriptive of available genomic data and should be interpreted cautiously in light of heterogeneous sampling over time.

**Figure 3:**
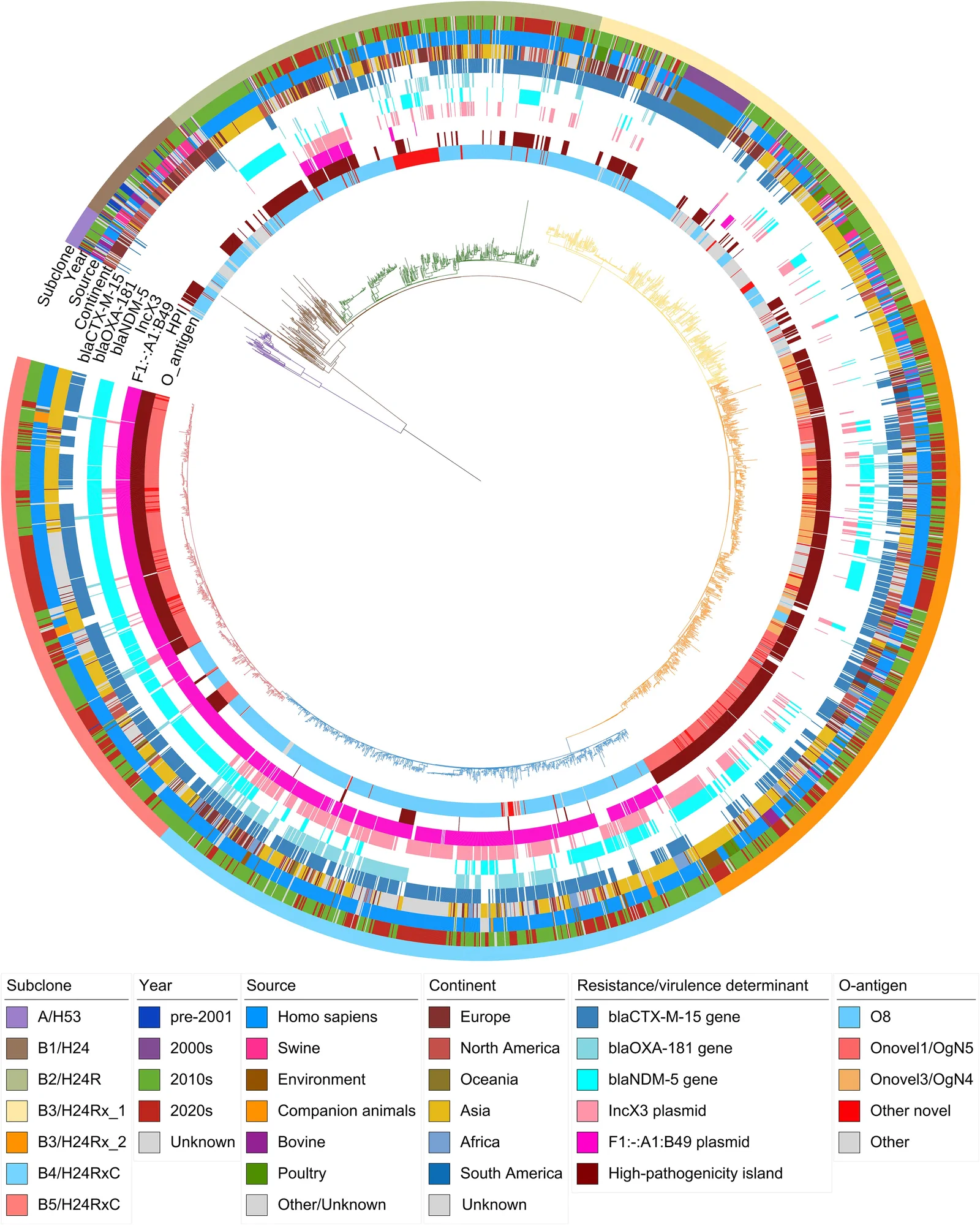
Phylogenetic structure and genomic features of *E. coli* ST410. Circular maximum-likelihood phylogenetic tree constructed from recombination-filtered core genome alignment of ST410 isolates. From the outermost to the innermost rings, annotations indicate subclone classification, year of collection, isolation source, continent of origin, presence of key resistance- and virulence-associated determinants, and O-antigen type. Colored blocks denote categorical metadata as defined in the legend. Annotations summarize available genomic and metadata information and should be interpreted cautiously in the context of heterogeneous sampling.

To understand the genetic evolution of ST410, we compared key genetic characteristics across the subclones, including serotype, resistance determinants, and virulence factors, in the context of the phylogenetic framework (Figs 2 and 3).

**Figure 4:**
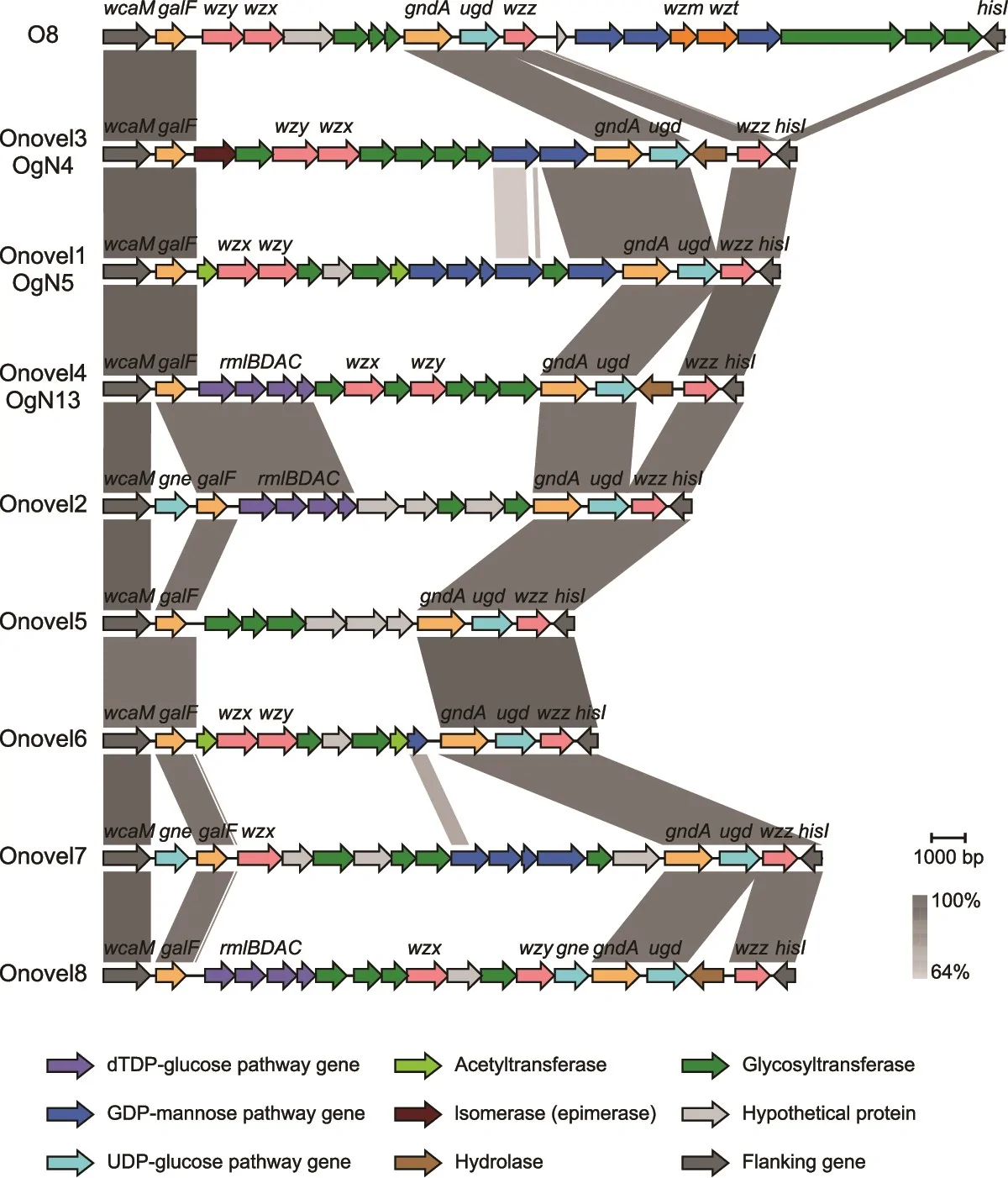
Sequence comparison of O8 and novel O-antigen gene clusters. O8 antigen (CP194961.1) was used as the reference. Onovel1, Onovel3, and Onovel4 were identical to previously reported OgN5 (LC177549.1), OgN4 (LC177548.1), and OgN13 (LC177550.1), respectively. Onovel2, Onovel5, Onovel6, Onovel7, and Onovel8 were characterized and designated in this study. Arrows indicate the open reading frames involved in O-antigen biosynthesis. Gray blocks between O-antigens represent homologous nucleotide sequences, with identity ranging from 64% to 100%. Branch lengths are proportional to the genetic distance.

Serotype analysis revealed extensive O-antigen diversity. Among 2077 isolates with identifiable O types, we detected 58 known O-antigen types. The most common was O8 (*n* = 1670), followed by O131 (*n* = 59), O76 (*n* = 45), and O132 (*n* = 41). The remaining 1357 isolates were untypable by SerotypeFinder, suggesting the presence of novel O-antigens. A random subset of these isolates was selected, and their O-antigens were characterized by identifying genes flanking the O-antigen gene clusters, resulting in the annotation and classification of 1023 isolates into eight distinct O-antigen types. Subsequent nucleotide BLAST analysis revealed that the most prevalent type (*n* = 740) showed high sequence identity to the previously reported OgN5 from pathogenic *E. coli* [56], also referred to as Onovel1 [23], followed by Onovel3 (*n* = 248), with smaller fractions for Onovel2 (*n* = 22), Onovel4 (*n* = 2), Onovel5 (*n* = 2), Onovel6 (*n* = 2), Onovel7 (*n* = 4), Onovel8 (*n* = 3), and other not-yet-classified O-antigens (*n* = 334). In addition to Onovel1, Onovel3 and Onovel4 corresponded closely to O-antigen gene clusters reported in pathogenic *E. coli* OgN4 and OgN13, respectively (Fig. 4). Moreover, we observed a clear pattern of antigenic differentiation between B4/H24RxC and B5/H24RxC with close phylogenetic relationship: the former was overwhelmingly O8-positive (679/737), whereas only a minority of B5/H24RxC (149/614) expressed O8, the majority of which expressed Onovel1 antigen (*n* = 411) and other novel types (*n* = 52) (Fig. 2A). This finding suggests that antigenic variation contributed to the immune evasion and success of the B5/H24RxC subclone. Within the B3/H24Rx subclone, we also noted a progressive shift: the earlier subclade B3/H24Rx_1 still had a substantial proportion of O8 (259/529) alongside novel antigens, whereas the more recent subclade B3/H24Rx_ 2 had almost completely lost O8 (22/807) in favor of novel O-antigens, including Onovel1 (*n* = 328), Onovel3 (*n* = 248), and other novel types (*n* = 115). Consistently, the overall prevalence of O8 within ST410 declined significantly over time (*P* < .013), whereas Onovel1 increased in frequency (*P* < .01) (Fig. 2B; Supplementary Fig. 9), mirroring the rise of B5/H24RxC and B3/H24Rx_2.

We examined chromosomal resistance mutations and acquired resistance genes to trace the stepwise evolution of multidrug resistance in ST410. Most ST410 genomes (3246/3434) harbored chromosomal mutations in QRDR of *gyrA*, *parC,* and/or *parE* genes conferring fluoroquinolone resistance. The prevalence of QRDR mutations was low in the early lineages A/H53 and B1/H24, but reached 100% in other subclones (Fig. 2C). For acquired resistance determinants, the ESBLs in ST410 were predominantly encoded by *bla*_CTX-M_ genes (2455/3434). *bla*_CTX-M-15_ was by far the dominant allele (*n* = 1998), followed by *bla*_CTX-M-55_ (*n* = 277). The prevalence of *bla*_CTX-M-15_ was low in the early lineages A/H53 and B1/H24, but increased progressively in more recent subclones. A majority of ST410 isolates (2143/3434) also carried at least one carbapenemase-encoding gene, including seven *bla*_NDM_, six *bla*_OXA_, two *bla*_IMP_, and three *bla*_KPC_ variants. The B4/H24RxC and B5/H24RxC subclones had particularly high carbapenemase prevalence (88.6% and 98%) compared to lower proportions in subclones A/H53, B1/H24, B2/H24R, and B3/H24Rx. Overall, carbapenemase occurrence in ST410 had risen steadily over the past two decades (*P* < .001; Fig. 2D and E). Among carbapenem resistance genes, *bla*_NDM-5_ was the most prevalent carbapenem resistance gene (*n* = 1330), followed by *bla*_OXA-181_ (*n* = 594). The *bla*_NDM-5_ carriage rate showed a clade-specific pattern: it was absent in A/H53 and B1/H24 isolates but universal in B5/H24RxC isolates (586/614), and within the B3/H24Rx subclone, *bla*_NDM-5_ was more common in B3/H24Rx_2 (307/807) than B3/H24Rx_1 (47/529). Temporal analysis indicated a steady increase in *bla*_NDM-5_ prevalence (*P* < .001). Conversely, *bla*_OXA-181_ gene and its derivatives *bla*_OXA-232_ and *bla*_OXA-484_ were mostly confined to the B4/H24RxC (*n* = 368) and B2/H24R (*n* = 137) and presented a decreasing prevalence over time. Moreover, plasmid replicon analysis showed that three IncF replicons (FII:1, FIA:1, and FIB:49) were predominantly positive in *bla*_NDM-5_-carrying B5/H24RxC (578/586) and *bla*_NDM-5_-carrying B4/H24RxC (205/240) isolates, but were rare or absent in the other subclones (Supplementary Data 1), which is consistent with *bla*_NDM-5_ being primarily located on an F1:A1:B49-type plasmid in these two subclones [23]. Altogether, our data show that ST410 has successively acquired resistance to multiple antibiotic classes in a stepwise fashion. Early quinolone-susceptible sublineages gave way to fluoroquinolone-resistant ones; then ESBL producers (*bla*_CTX-M-15_) proliferated; and ultimately carbapenemase-producing subclones (especially those with *bla*_NDM-5_) became dominant. The B5/H24RxC subclone now represents a worst-case scenario of this trajectory, being resistant to virtually all major antibiotic classes—raising serious concerns for future treatment of ST410 infections. We also infer from the genomic data that the ST410 lineage likely originated in the A/H53 subclone and sequentially branched into B1/H24, followed by B2/H24R, B3/H24Rx, and B4/H24RxC, with B5/H24RxC as a recently emerged offshoot of B4/H24RxC.

We surveyed virulence-associated genes, focusing on the yersiniabactin-encoding HPI. Overall, over half of ST410 isolates (1746/3434) carried a complete HPI. However, HPI prevalence varied widely by subclone. It was relatively high in subclone B5/H24RxC (460/614) but very low in B4/H24RxC (47/737). Within B3/H24Rx, the HPI was only seen in a minority of B3/H24R_1 (132/529) but was prevalent in the expanding B3/H24R_2 (766/807), consistent with clonal enrichment of this virulence locus. Over time, HPI carriage in ST410 isolates increased significantly (*P* < .001), indicating positive selection during recent evolution. In parallel, HPI and *bla*_NDM-5_ expanded together: co-occurrence rate reached 72.3% in B5/H24RxC and 37.2% in B3/H24Rx_2, higher than in any other subclone (e.g. B4/H24RxC, 0.8%; B3/H24Rx_1, 1.5%), and showed an increase over time (*P* < .001). These markedly higher rates in the very groups undergoing rapid expansion point to a synergistic advantage—strains combining HPI with *bla*_NDM-5_ likely enjoy improved fitness under host-like metal limitation and antibiotic exposure—helping to fuel the rise of B5/H24RxC and B3/H24Rx_2. To test whether this pattern is unique to ST410 or part of a broader trend, we extended our analysis to all available *E. coli* genomes in GenBank (*n* = 346316), and found that the combined HPI prevalence (58069/81674) among the top 20 ExPEC lineages (ST131, ST69, ST95, ST73, ST648, ST405, ST410, ST167, and others) [57] was significantly higher than that in other *E. coli* lineages (52539/264642) (*P* < .0001; Fig. 2F). Moreover, across the entire *E. coli* dataset, *bla*_NDM_ carriage was more common in HPI-positive isolates (6099/110608) than in HPI-negative isolates (7355/235708) (*P* < .0001; Fig. 2G), further supporting a model of co-evolution between resistance and virulence in *E. coli*.

In summary, our genomic analysis of ST410 reveals a stepwise evolutionary path toward a high resistance–high virulence profile. This path involved (i) accumulation of resistance mutations and genes (from fluoroquinolone resistance to ESBLs to carbapenemases), (ii) antigenic shifts presumably aiding immune and/or bacteriophage evasion (replacement of O8 with novel O-antigens in emerging subclades), and (iii) acquisition of a major virulence locus (HPI) concurrent with carbapenem resistance gene (*bla*_NDM-5_). The convergence of *bla*_NDM-5_ and HPI in the recent subclones B5/H24RxC and B3/H24Rx_2 is particularly alarming, as it produces bacteria that are both difficult to treat and potentially more aggressive in infection.

### HPI-mediated iron uptake drives *in vivo* fitness and virulence of ST410

**Figure 5:**
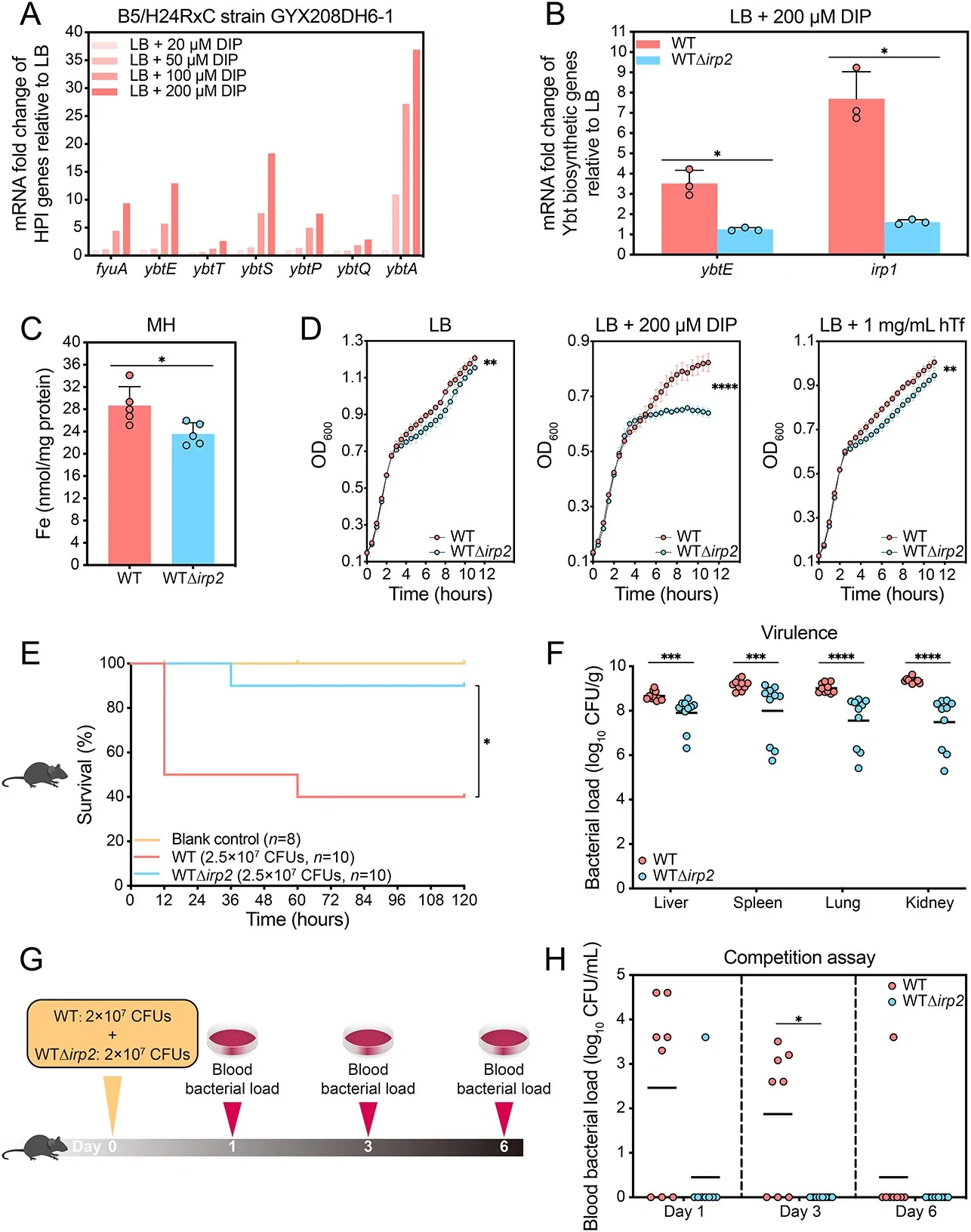
HPI functions as a siderophore system that promotes iron acquisition, bacterial fitness, and virulence. (A) Relative expression levels of HPI-associated genes in the B5/H24RxC strain GYX208DH6-1 under iron-depleted conditions (LB + DIP) compared with standard LB medium. (B) Relative expression levels of yersiniabactin (ybt) biosynthesis genes in the B3/H24Rx_2 strain AHM7C101I (WT) and its *irp2* deletion mutant (WTΔ*irp2*) under iron-depleted (LB + DIP) versus standard LB conditions. Gene expression was quantified by RT-qPCR and normalized to 16S rRNA. Data are shown as mean ± SD from three biological replicates. (C) Intracellular iron levels of WT and WTΔ*irp2* strains cultured in MH broth, measured by ICP-OES (mean ± SD, *n* = 5). (D) Growth curves of WT and WTΔ*irp2* strains under normal (LB) and iron-depleted conditions (LB + DIP or LB + hTf). OD_600_ was recorded every 30 min (mean ± SD, *n* = 4). (E) Survival of mice following infection with WT or WTΔ*irp2* strains (*n* = 10 per group). (F) Bacterial load in major organs of mice infected with WT or WTΔ*irp2* strains (mean ± SD, *n* = 10 per group). (G, H) *In vivo* competition assays between WT and WTΔ*irp2* strains. Mice (*n* = 8) were infected with a 1:1 mixture, and bacterial load in the tail vein blood was quantified at the indicated time points.

HPI encodes the yersiniabactin siderophore system, which is well known to increase iron acquisition and virulence in bacteria [27, 28]. The strong genomic association between HPI and *bla*_NDM-5_, coupled with the rising dominance of HPI-positive subclones, led us to hypothesize that HPI provides a fitness advantage to ST410, especially under host-like conditions (e.g. iron limitation). We first confirmed that the HPI in ST410 is functional and responsive to iron availability. Using RT-qPCR, we measured expression of HPI genes in a B5/H24RxC subclone strain (GYX208DH6–1, carrying HPI and *bla*_NDM-5_) [40] under iron-replete versus iron-depleted conditions. In iron-chelated media (LB + 2,2′-dipyridyl, DIP), the strain showed dose-dependent upregulation of HPI genes compared to growth in standard LB (Fig. 5A). Higher DIP concentrations (greater iron stress) induced progressively stronger expression of genes in the yersiniabactin biosynthesis and transport operons, indicating that the iron-regulated promoter system of HPI is active in ST410. Thus, ST410 can sense iron limitation and respond by upregulating yersiniabactin production, presumably to scavenge iron.

To directly validate the role of yersiniabactin in iron uptake, we created an *irp2* knockout mutant, which encodes a non-ribosomal peptide synthetase essential for yersiniabactin biosynthesis, in ST410 and compared it to the wild type. Because the B5/H24RxC subclone strain GYX208DH6–1 was refractory to genetic manipulation due to pSC101-like plasmid incompatibility, we chose a B3/H24Rx_2 subclone strain AHM7C101I [39], which also carried HPI and *bla*_NDM-5_. The HPI sequence in AHM7C101I was mostly identical to that of the B5/H24RxC strain, differing by only two synonymous SNPs, making it a suitable surrogate. We confirmed by RT-qPCR that deleting *irp2* abolished yersiniabactin production. The *ybtE* and *irp1* genes, which encode synthetase in the yersiniabactin biosynthesis pathway and work together with *irp2* to synthesize this iron-chelating siderophore [58], were measured. In an iron-depleted medium (200 μM DIP), the WT strain showed strong induction of other HPI biosynthetic genes, as *ybtE* and *irp1* increased 3.5-fold and 7.7-fold, respectively, relative to normal conditions, whereas the *irp2*-knockout mutant (WTΔ*irp2*) showed almost no induction of these genes (1.2-fold, 1.6-fold increased) (*P* < .05; Fig. 5B). This indicates the Δ*irp2* mutant cannot effectively produce yersiniabactin or activate the HPI system under iron stress.

The HPI-deficient mutant had measurably lower intracellular iron content than the wild type. Using ICP-OES, we found that in the MH medium, the WT strain accumulated significantly more iron inside the cells (28.7 nM) compared to the isogenic *irp2* mutant (23.6 nM) (*P* < .05; Fig. 5C), providing direct evidence that HPI enhanced iron uptake capacity in ST410. Given that iron availability is critical for bacterial growth, we next examined whether HPI-enhanced iron uptake confers a growth advantage. Indeed, when we compared the growth of WT and WTΔ*irp2* under either iron-depleted condition (LB + 200 μM DIP and LB + 1 mg/ml human transferrin, hTf) or normal condition (standard LB), the WT grew markedly faster and to a higher density than the mutant, with the WTΔ*irp2* strain showing particularly restricted growth in the iron-depleted condition (*P* < .01; Fig. 5D). These data show that HPI-mediated iron acquisition improves the growth fitness of ST410, particularly under iron-limited conditions that mimic the host environment.

We assessed whether the contribution of HPI to iron uptake and growth translates into higher virulence *in vivo*. Using the mouse intraperitoneal infection model, we revealed that mice infected with the WT (HPI^+^) strain experienced significantly higher mortality than those infected with the WTΔ*irp2* (HPI^−^) strain (*P* < .05; Fig. 5E). The 50% lethal dose (LD_50_) of the WT (HPI^+^) strain was ~2.5 × 10^7^ CFU per mouse, whereas that of the WTΔ*irp2* (HPI^−^) strain exceeded 1 × 10^8^ CFU per mouse. Furthermore, in a separate set of infected mice without antibiotic treatment, the bacterial burdens in organs were substantially higher for WT than for WTΔ*irp2*. At 12 h postinfection, median CFU counts of WT in the liver (*P* < .001), spleen (*P* < .001), lungs (*P* < .0001), and kidneys (*P* < .0001) were all significantly greater (by 1–2 log_10_) than counts of the mutant (Fig. 5F). In the coinfection assay, when mice were inoculated with an equal mixture of WT and WTΔ*irp2* (2 × 10^7^ CFUs each, nonlethal amounts), the WT accounted for the majority of recovered CFUs, significantly outnumbering the WTΔ*irp2* mutant by orders of magnitude at 3 days postinfection (*P* < .05), and required six days to be nearly cleared by the host, whereas the WTΔ*irp2* mutant was almost eliminated within 1 day postinfection (Fig. 5G and H), suggesting the HPI^+^ strain out-competed the HPI^−^ strain inside the host and confirming that HPI provides a tangible fitness benefit during infection.

Collectively, these findings establish HPI as a significant virulence and fitness factor in ST410. By enabling better iron uptake, HPI allows ST410 to grow faster in iron-restricted host conditions and to achieve higher densities in tissues, leading to more severe infection outcomes. This helps explain why HPI-positive clones of ST410 (like B5/H24RxC and B3/H24Rx_2) have surged: HPI acquisition likely gave them a selective advantage in causing invasive infections. More broadly, it suggests that the yersiniabactin system is a key evolutionary adaptation that can boost the pandemic potential of *E. coli* lineages. This may also clarify why HPI tends to be associated with the most successful ExPEC clones, as we observed in the global data.

### HPI-mediated zinc acquisition potentiates NDM-5 and undermines carbapenem efficacy

**Figure 6:**
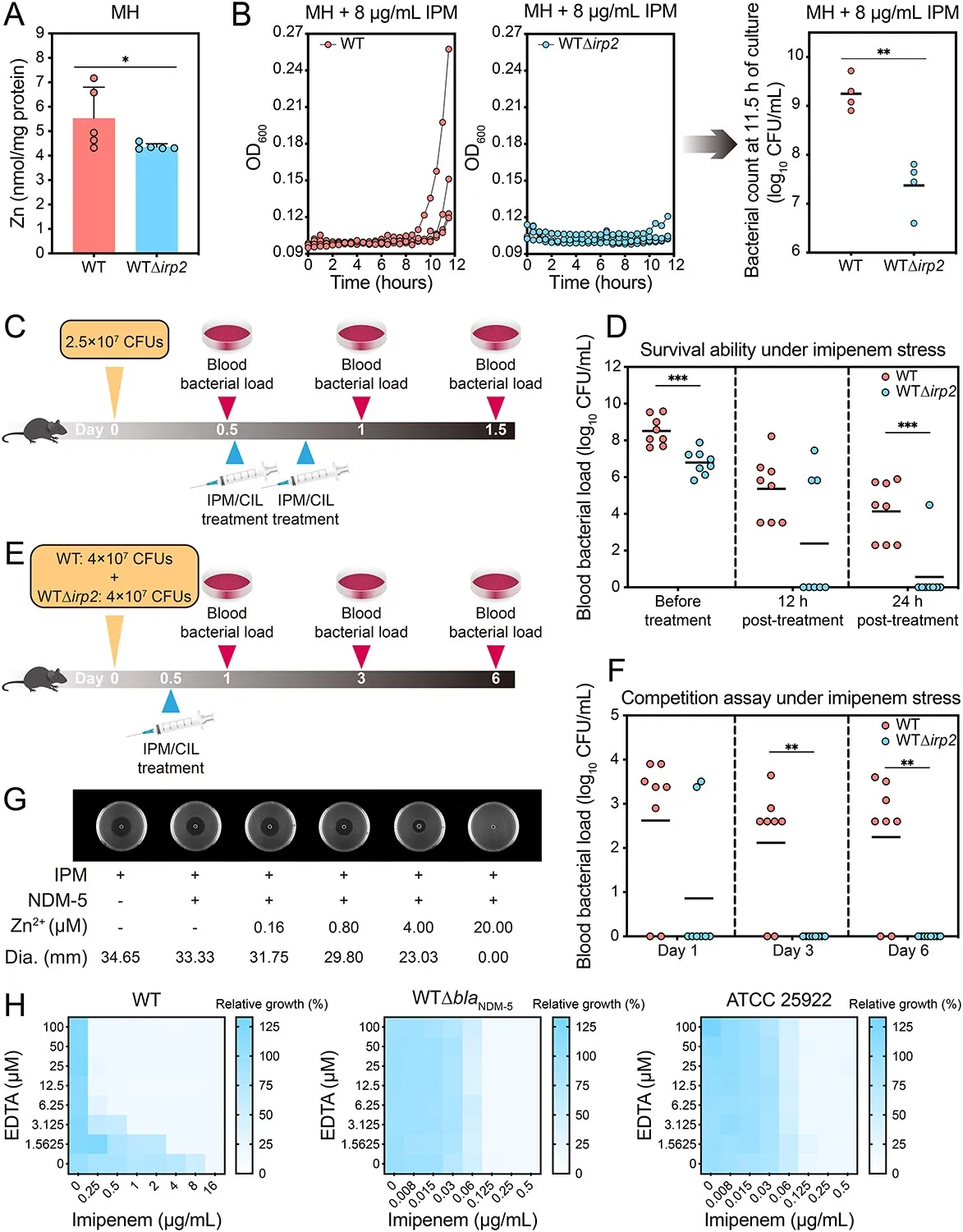
HPI-mediated zinc acquisition enhances NDM-5 β-lactamase activity and bacterial survival under carbapenem pressure. (A) Intracellular zinc levels of WT and WTΔ*irp2* strains grown in MH broth, measured by ICP-OES (mean ± SD, *n* = 5). (B) Growth curves of WT and WTΔ*irp2* strains under sub-inhibitory imipenem exposure (MH + 8 μg/ml IPM). OD_600_ was recorded every 30 min, and viable counts were determined after 11.5 h (mean ± SD, *n* = 4). (C, D) *In vivo* survival of WT and WTΔ*irp2* strains during imipenem treatment. Mice (*n* = 8 per group) were infected with WT or WTΔ*irp2* strains. Bacterial load in the tail vein blood was quantified 12 h post-infection (before treatment). Mice then received imipenem/cilastatin (50 mg/kg IPM/CIL) twice at a 6-h interval, followed by bacterial quantification at 12 h and 24 h post-treatment. (E, F) Competitive infection assays under imipenem stress. Mice (*n* = 8) were infected with a 1:1 mixture of WT and WTΔ*irp2* strains and treated with IPM/CIL (50 mg/kg) at 12 h post-infection. Blood bacterial load was quantified at the indicated time points. (G) Hydrolysis activity of NDM-5 against imipenem at increasing Zn^2+^ concentrations. Reaction mixtures containing 768 μg/ml imipenem and 49 μg/ml purified NDM-5 protein were incubated with increasing concentrations of ZnCl_2_ (total volume, 50 μL) at 37°C for 30 min. Residual imipenem activity was assessed using inhibitory zone assays against *E. coli* ATCC 25922. (H) Checkerboard assays of EDTA–imipenem combinations showing the zinc dependence of NDM-5-mediated resistance. Growth of NDM-5-producing (WT) and non-producing strains (WTΔ*bla*_NDM-5_ and ATCC 25922) was tested in MH broth containing increasing EDTA concentrations (0–100 μM). Color intensity reflects normalized bacterial growth relative to untreated controls.

Recent studies have shown that yersiniabactin can bind other divalent metal ions such as nickel, copper, and zinc [59–61]. Yersiniabactin has been implicated in overcoming zinc sequestration defenses during *Yersinia pestis* infections [62]. This raises a possibility, because NDM-5 is a zinc-dependent metallo-β-lactamase requiring two Zn^2+^ ions in its active site for maximal activity [63], HPI might also function as a “zincophore” for ST410, increasing intracellular Zn^2+^ supply and thereby enhancing NDM-5-mediated carbapenem resistance. To test this hypothesis, we first measured the intracellular zinc content of the WT versus WTΔ*irp2* strains using ICP-OES. Even in standard MH broth, the HPI-positive WT maintained a significantly higher Zn concentration (5.5 nM) than the mutant (4.4 nM) (*P* < .05, Fig. 6A), confirming that HPI not only enhances iron uptake in ST410 but also improves zinc ion acquisition.

To determine whether this extra zinc translates into higher carbapenem resistance, we used standard broth minimum inhibitory concentration (MIC) testing and revealed no difference in the imipenem MICs (16 μg/ml) between WT and WTΔ*irp2*. However, when we challenged the strains with 1/2 imipenem MICs (8 μg/ml), we observed a clear divergence in the growth curve. The WT culture, after an initial suppression, began regrowing after ~10 h, whereas the WTΔ*irp2* culture remained growth-restricted throughout the 12-h period (Fig. 6B). By the end of the assay (11.5 h), the WT achieved 9.2 log_10_ CFU/ml, whereas the WTΔ*irp2* stayed around 7.4 log_10_ CFU/ml (*P* < .01). These findings suggest that HPI enhances bacterial survival under carbapenem pressure. We then evaluated whether this phenomenon holds true *in vivo* using our mouse infection model with imipenem treatment (Fig. 6C). Mice were infected with either WT or WTΔ*irp2* and, after 12 h of infection, given a single dose of imipenem to mimic clinical therapy. Prior to drug administration, consistent with earlier virulence results, the blood bacterial load was significantly higher in WT-infected mice (8.5 log_10_ CFU/ml) than in mutant-infected mice (6.5 log_10_ CFU/ml) (*P* < .001; Fig. 6D). After 12 h of imipenem treatment, both groups showed a drop in bacterial load: WT decreased to 5.4 log_10_, WTΔ*irp2* to 2.4 log_10_. However, after 24 h, a difference emerged: the WT infection persisted with only a slight further decrease (4.1 log_10_ CFU/ml), whereas the Δ*irp2* infection was almost completely cleared (0.6 log_10_ CFU/ml) (*P* < .001). These results suggest that HPI undermines carbapenem efficacy; even in the presence of strong host immune clearance and high systemic imipenem exposure, HPI enables bacterial persistence under dual host–drug pressures. Further, competitive mixed infections under imipenem treatment confirmed that the WT strain overwhelmingly outcompeted the WTΔ*irp2* strain (Days 3 and 6: *P* < .01) (Fig. 6E and F), as the HPI^+^ strain dominated the bloodstream population throughout the 6-day course, whereas the WTΔ*irp2* was largely suppressed from the very beginning. These *in vivo* data provide evidence that HPI diminishes the efficacy of carbapenem treatment against NDM-5-producing ST410, leading to treatment failure in the case of HPI-positive infection.

To elucidate the mechanistic basis for HPI-mediated carbapenem resistance, we hypothesized that if HPI allows the bacteria to accumulate more Zn^2+^, NDM-5 could operate at higher efficiency, breaking down imipenem more rapidly. We tested NDM-5 activity *in vitro* by observing imipenem hydrolysis in the presence of varying zinc concentrations. Purified NDM-5 enzyme (Supplementary Fig. 10) on its own caused a slight reduction in the efficacy of imipenem, as the inhibition zone in an agar diffusion assay shrank from 34.65 mm without NDM to 33.33 mm with NDM-5. As we incrementally increased the Zn^2+^ concentration from 0.16 μM up to 20 μM in the NDM-5 + imipenem mixture, we observed a stepwise decrease in the inhibition zone diameter—from 31.75 mm down to 0 mm (Fig. 6G). This dose-dependent enhancement confirms that the carbapenemase activity of NDM-5 is strongly zinc-limited: more zinc means more active enzyme and thus greater resistance. Conversely, when we created zinc-poor conditions using EDTA as a chelator, we observed the mirror image: as EDTA levels increased, the imipenem MIC for the NDM-5-positive strain dropped, indicating more susceptible due to diminished NDM function. For example, adding EDTA lowered the effective imipenem resistance of WT to the range of the NDM-negative controls. In contrast, EDTA had no effect on the imipenem MIC of the NDM-5-negative strains (WT∆*bla*_NDM-5_ and ATCC 25922), which remained constant (Fig. 6H).

Together, these data establish a mechanistic link between HPI and NDM-5 in ST410: HPI increases intracellular Zn^2+^, which boosts NDM-5 catalytic activity, leaving MICs unchanged yet promoting survival and regrowth under sub-MIC carbapenem exposure and during therapy *in vivo*. Chelation (EDTA) reverses this advantage, and specificity controls exclude serine carbapenemases, pinpointing a zinc-dependent effect. This HPI–NDM-5 axis explains the frequent co-occurrence of the two loci in expanding subclones and highlights metal homeostasis as a tractable adjunct target to restore carbapenem efficacy.

## Discussion

Our findings have shown that a multidrug-resistant *E. coli* clone can evade typical resistance–fitness trade-offs through a synergistic virulence mechanism. ST410 has recently emerged as a high-risk lineage with global spread and extensive drug resistance [20–23, 64]. Resistance acquisition is a principal driver, as illustrated by pandemic ST131 with CTX-M-encoding plasmids [65]. Indeed, ST410 followed a similar stepwise path—from quinolone-resistance mutations to ESBL production and ultimately carbapenemase acquisition—consistent with selection under antibiotic pressure. Although resistance often imposes fitness or virulence costs [66], ST410 appears to circumvent these trade-offs: its most successful subclones display a high-resistance and high-virulence phenotype, akin to hypervirulent carbapenem-resistant *K. pneumoniae* ST11 [67]. In contrast to *Klebsiella*, where convergence is typically mediated by virulence plasmids, ST410 achieves this through a chromosomally encoded HPI.

We found HPI to be widespread in ST410 and particularly enriched in the dominant subclades B5/H24RxC and B3/H24Rx_2. This suggests HPI has been positively selected in the recent evolution of ST410. The known function of HPI is to improve iron scavenging in iron-starved host environments, thereby enhancing bacterial growth and invasiveness. Indeed, we confirmed that HPI provides ST410 a significant advantage in iron-limited conditions and increases its virulence *in vivo*. Given that iron sequestration is a central component of host nutritional immunity [68, 69], a superior iron acquisition system like yersiniabactin can tip the scales in favor of the bacterium during infection. The rising prevalence of HPI in ST410 over time implies that this virulence factor has contributed substantially to the clinical and ecological success of this clone.

A central finding of this study is the functional synergy between HPI and *bla*_NDM-5_. We provide experimental evidence that a virulence determinant can potentiate antibiotic resistance in a clinically relevant setting. Thus, we move beyond genomic co-occurrence to a causal link: the metal uptake function of a siderophore system elevates the efficacy of a resistance enzyme. This virulence–resistance coupling helps explain why HPI and *bla*_NDM-5_ co-segregate in expanding ST410 subclades and illustrates how pathogens can become simultaneously more aggressive and harder to treat. Similar convergence has been described in *K. pneumoniae* (e.g. ST11 variants combining hypervirulence and carbapenem resistance) [70, 71]; however, by using isogenic mutants and targeted perturbations, our work pinpoints the direction of effect—HPI-derived Zn^2+^directly augments NDM-5 hydrolysis—showing that the success of ST410 reflects interplay between loci rather than simple additive accumulation. Plasmid replicon analysis further suggests that *bla*_NDM-5_ in B5/H24RxC is likely carried by an F1:A1:B49-type plasmid, the backbone of which has been reported in our previous study to lack a complete conjugative transfer region, suggesting limited or absent self-transmissibility [40]. Such a genetic configuration is consistent with reduced plasmid-associated fitness costs and may favor stable vertical inheritance of *bla*_NDM-5_ within B5/H24Rx. Together with chromosomally encoded HPI, this combination may contribute to the clonal expansion of B5/H24RxC under combined antibiotic and metal-limited selective pressures. Collectively, these findings provide a mechanistic framework that helps explain the previous report of the increased resistance and virulence in this subclone and its outbreak in hospitals [23].

These findings have important clinical and ecological implications. ST410 carrying HPI and *bla*_NDM-5_ pose a two-pronged threat—simultaneously highly virulent and highly drug-resistant. *In vivo*, carbapenem therapy underperformed because HPI-mediated Zn^2+^ supply potentiated NDM-5 and enabled survival/regrowth under treatment. Clinicians should anticipate atypical responses to carbapenems in such cases and consider earlier escalation to combination or alternative therapies. Broadly limiting essential metals such as iron or zinc, especially in physiologically metal-limited environments like the gut, would impact commensal microbiota and therefore is unlikely to be feasible. Instead, our findings support pathogen-targeted approaches. One such strategy is the use of antibiotics that exploit siderophore-mediated uptake pathways (e.g. cefiderocol) [72], which selectively leverage bacterial iron acquisition systems rather than globally depriving the host or microbiome of metals; analogously, antibiotics combined with zinc-sequestering adjuvants that restrict zinc availability to metallo-β-lactamases merit further investigation for HPI-positive, NDM-producing infections. From an ecological perspective, the success of ST410 reflects the evolutionary adaptation to heterogeneous and resource-limited environments (e.g. iron-limited environments), where survival and competitiveness are shaped by multiple interacting selective pressures. The acquisition of HPI and its functional coupling to *bla*_NDM-5_ suggest that metal acquisition systems can act as ecological keystone traits, linking nutrient scavenging, antibiotic resistance, and competitive fitness. Such traits are expected to be favored not only during infection but also across host-associated and external reservoirs, as exemplified by outbreak-associated dissemination in veterinary settings and wastewater systems [18, 24, 73, 74], where metal scarcity and antibiotic residues co-occur. Together, our findings connect molecular mechanisms to ecological fitness, illustrating how environmental constraints and ecological selection can drive the evolution and global dissemination of multidrug-resistant high-risk clones.

Beyond the HPI–NDM axis, O-antigen diversification likely contributes to the expansion of dominant subclones: B5/H24RxC and B3/H24Rx_2 predominantly express novel O-antigens, whereas O8 declines, a pattern consistent with phage-mediated selection that reduces susceptibility to O8-targeting phages and drives immune evasion through antigenic variation to facilitate persistence and spread [75]. These shifts, together with HPI-driven metal acquisition and NDM-mediated resistance, underscore the need for surveillance that tracks not only resistance genes but also virulence loci (e.g. HPI) and surface-antigen changes. Early detection of HPI-positive, NDM-producing *E. coli*—particularly lineages carrying novel O-antigens—can flag highly fit clones capable of dissemination even under limited antibiotic pressure, informing infection control and risk assessment.

Despite the breadth of this work, several limitations remain. First, the genomic dataset depends on publicly available sequences and may be affected by geographic and clinical sampling biases. Additionally, genome availability has increased over time, particularly in recent years, which may influence the apparent emergence or frequency of specific strains or genetic features. Earlier time points with limited genome availability should therefore be interpreted with caution, as absence or low frequency may reflect incomplete data rather than true absence. As a result, representation in public databases should not be interpreted as a direct proxy for epidemiological prevalence or population-level success, despite our inclusion of diverse isolates. Instead, this dataset provides a valuable resource for exploring broad patterns of lineage representation and the co-occurrence of genomic features. In addition, our analyses do not explicitly account for phylogenetic nonindependence among isolates; consequently, some observed lineage–trait associations may partly reflect shared evolutionary history and should be interpreted with appropriate caution. Second, genetic manipulation of B5/H24RxC was not feasible with current tools; functional assays therefore used a B3/H24Rx_2 proxy with near-identical HPI and resistance loci. Direct validation in a B5/H24RxC background—via improved genome-editing methods—would be ideal. Third, even though we implicate zinc in potentiating NDM-5, the molecular handling of Zn^2+^ by yersiniabactin—including trafficking and delivery to periplasmic targets—remains unresolved; structural analyses and live-cell metal imaging could clarify this mechanism.

**Figure 7:**
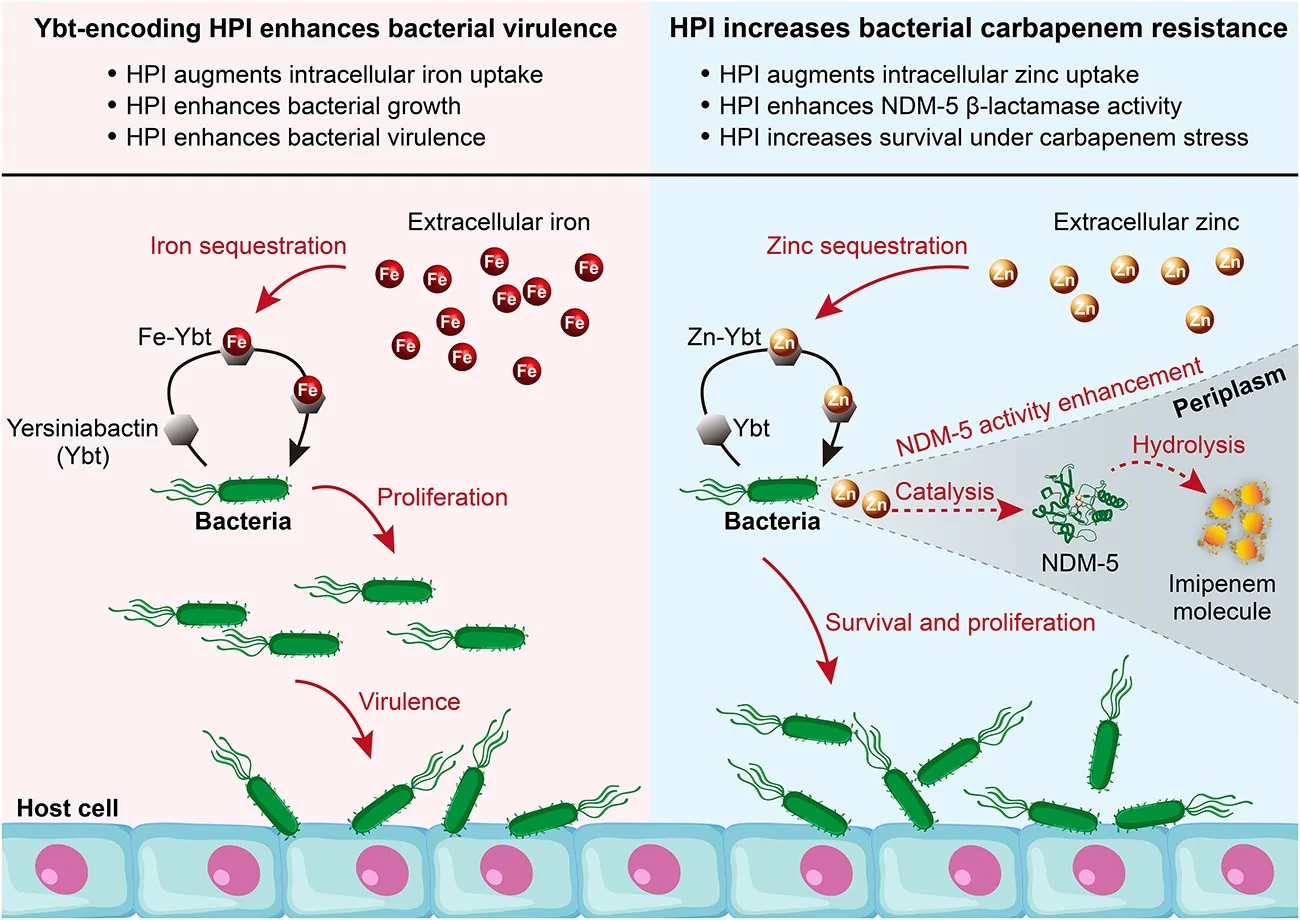
Proposed model illustrating the role of HPI in *E. coli* ST410. HPI-mediated metal acquisition enhances bacterial virulence and promotes survival under carbapenem exposure by increasing the zinc-dependent NDM-5 activity.

In conclusion, this work dissects the ecological, genomic, and mechanistic foundations of the rise of *E. coli* ST410. We identify B3/H24Rx_2 as an emerging high-risk subclone mirroring B5/H24RxC, indicating at least two lineages that have convergently evolved a hyper-resistant, virulent phenotype and are increasing in prevalence. We further elucidate a co-evolutionary HPI–NDM-5 axis: HPI-driven metal acquisition potentiates NDM-5, thereby coupling enhanced virulence with heightened resistance (Fig. 7). This virulence–resistance synergy reframes how pathogen success should be understood in the antibiotic era and highlights metal homeostasis as a tractable vulnerability for intervention. More broadly, our findings emphasize the need for integrative surveillance and control strategies that consider both ecological context and evolutionary dynamics as highly fit bacterial clones continue to emerge.

## Supplementary Material

Supplementary_material_wrag086

## Data Availability

All data generated or analyzed during this study are included in this published article and its supplementary information files. Scripts and data required to reproduce the figures in the manuscript are available at https://github.com/WanyunHe/EC-ST410_project.
