# Supplementary material for: High-pathogenicity island enhances NDM-5 activity and drives global spread of Escherichia coli ST410
Source: ISME J. 2026 Apr 11;20(1):wrag086. doi: 10.1093/ismejo/wrag086 (PMC13502196; doi:10.1093/ismejo/wrag086)
Supplement: Supplementary file 1 [file supplementary_material_wrag086.zip › Supplementary_Material_wrag086.docx]

**Supplementary Material for:**

**High-pathogenicity island enhances NDM-5 activity and drives global spread of *E******scherichia coli* ST410**

Wanyun He^1†^, Jieying Tu^1†^, Zhongpeng Cai^1^, Zixuan Wang^1^, Chuying Liang^1^, Yingbo Gao^1^, Guolong Gao^1^, Luchao Lv^1^, Jun Yang^3^, Yi-Yun Liu^1^, Jianzhong Shen^2^*, Yang Wang^2^*, Jian-Hua Liu^1^*

^1^ State Key Laboratory for Animal Disease Control and Prevention, Key Laboratory of Zoonosis of Ministry of Agricultural and Rural Affairs, College of Veterinary Medicine, South China Agricultural University, Guangzhou 510642, China.

^2^ National Key Laboratory of Veterinary Public Health Security, College of Veterinary Medicine, China Agricultural University, Beijing, China.

^3^ Guangdong Provincial Key Laboratory of Pharmaceutical Bioactive Substances, School of Basic Medical Sciences, Guangdong Pharmaceutical University, Guangzhou 510006, China.

^†^ These authors contributed equally to this work.

* Corresponding authors. Jianzhong Shen (sjz@cau.edu.cn); Yang Wang (wangyang@cau.edu.cn); Jian-Hua Liu (jhliu21@163.com)

**Materials included:**

Supplementary Figures 1 to 10

Supplementary Tables 1 to 2

Legends for Supplementary Data 1 to 4

Supplementary Data 1 to 4 are provided as .xlsx files for download

**Supplementary Figure 1.** Global geographical distribution of A/H53 isolates. Countries from which isolates have been identified are shaded in grey. Symbols indicate isolation sources, as annotated in the legend, and are placed at approximate geographic locations for visualization purposes. All distributions are descriptive of publicly available metadata information and should be interpreted cautiously in light of heterogeneous sampling.

**Supplementary Figure 2.** Global geographical distribution of B1/H24 isolates. Countries from which isolates have been identified are shaded in grey. Symbols indicate isolation sources, as annotated in the legend, and are placed at approximate geographic locations for visualization purposes. All distributions are descriptive of publicly available metadata information and should be interpreted cautiously in light of heterogeneous sampling.

**Supplementary Figure 3.** Global geographical distribution of B2/H24R isolates. Countries from which isolates have been identified are shaded in grey. Symbols indicate isolation sources, as annotated in the legend, and are placed at approximate geographic locations for visualization purposes. All distributions are descriptive of publicly available metadata information and should be interpreted cautiously in light of heterogeneous sampling.

**Supplementary Figure 4.** Global geographical distribution of B3/H24Rx isolates. Countries from which isolates have been identified are shaded in grey. Symbols indicate isolation sources, as annotated in the legend, and are placed at approximate geographic locations for visualization purposes. All distributions are descriptive of publicly available metadata information and should be interpreted cautiously in light of heterogeneous sampling.

**Supplementary Figure 5.** Global geographical distribution of B4/H24RxC isolates. Countries from which isolates have been identified are shaded in grey. Symbols indicate isolation sources, as annotated in the legend, and are placed at approximate geographic locations for visualization purposes. All distributions are descriptive of publicly available metadata information and should be interpreted cautiously in light of heterogeneous sampling.

**Supplementary Figure 6.** Global geographical distribution of B5/H24RxC isolates. Countries from which isolates have been identified are shaded in grey. Symbols indicate isolation sources, as annotated in the legend, and are placed at approximate geographic locations for visualization purposes. All distributions are descriptive of publicly available metadata information and should be interpreted cautiously in light of heterogeneous sampling.

**Supplementary Figure 7.** Global geographical distribution of B3/H24Rx_1 and B3/H24Rx_2 isolates. Countries from which isolates have been identified are shaded in grey. Symbols indicate isolation sources, as annotated in the legend, and are placed at approximate geographic locations for visualization purposes. All distributions are descriptive of publicly available metadata information and should be interpreted cautiously in light of heterogeneous sampling.

**Supplementary Figure 8.** Core genome-based pairwise SNP distance distributions among ST410 subclones. Violin plots depict the overall distribution of pairwise SNP distances within each subclone based on recombination-filtered core genome alignments. White dots indicate the median SNP distance for each subclone, with corresponding values shown.

**Supplementary Figure 9.** Temporal trends in the relative abundance of O8 and Onovel1 antigens in ST410 over time (normalized to 0–1). Shaded areas indicate 95% confidence intervals. Pearson correlation analysis with linear regression was applied to assess temporal associations. The coefficient of determination (*R*^2^) was calculated using linear models. Statistical significance was evaluated using two-sided Pearson correlation tests.

**Supplementary Figure 10.** SDS-PAGE analysis of purified NDM-5 protein. Lane M, protein maker; lane F, flow-through fraction; lanes E1 to E6, eluates obtained with 0, 100, 200, 300, 400, and 500 mM imidazole, respectively. Molecular weights (kDa) are indicated on the left. The purified NDM-5 protein (>25 kDa, arrow) was predominantly eluted in the 200 mM imidazole fraction, indicating successful purification.

**Supplementary Table 1.** Bacterial strains and plasmids used in this study.

| Strains | Genotype | Reference |
| --- | --- | --- |
| GYX208DH6-1 | *bla*_NDM-5_-positive F1:A1:B49 plasmid, multidrug resistance, HPI positive | [1] |
| AHM7C101I (WT) | *bla*_NDM-5_-positive IncX3 plasmid, multidrug resistance, HPI positive | [2] |
| WT∆*irp2* | *bla*_NDM-5_-positive IncX3 plasmid, multidrug resistance, HPI deficient | This study |
| WT∆*bla*_NDM-5_ | Carbapenem susceptible | This study |
| ATCC 25922 | Antibiotic-susceptible reference control | This study |
| DH5α | F^-^, Ф­80(*lacZ*∆M15), ∆(*lacZYA*-argF)U169, *endA*1, *recA*1, *hsdR*17(rK^-^, mK^+^), *supE*44, λ^‑^, *thi*1, *gyrA*96, *relA*1, *phoA* | Beijing Tsingke Biotech |
| BL21(DE3) | F^-^, *ompT*, *hsdS*_β_(rB^-^, mB^-^)*gal*, *dcm,* λ(DE3) | Beijing Tsingke Biotech |
| pCasKP | Cas9 expression plasmid, *araBAD* promoter, chloromycin resistance, temperature-sensitive origin of replication | [3] |
| pSGKP | Single guide RNA expression plasmid, constitutive promoter, rifampicin resistance, compatible with pCasKP, *sacB* counter-selection marker | [3] |
| pET28a | T7 promoter expression vector, N- and C-terminal His6-tag, kanamycin resistance, *lacI* gene, ColE1 origin of replication | This study |

**Supplementary Table 2.** Primers used in this study.

| Primers | Sequences (5'-3') |
| --- | --- |
| fyuA-F | TCTTACCCTGGTCGCCAAAC |
| fyuA-R | CAGTGACCTGACCTGGCATT |
| irp1-F | TTGGGCGACAGTGAAGGAAG |
| irp1-R | GTGGGAAACGCTGCATCAAG |
| ybtE-F | GTCCAGCCGGGTAAAACAGA |
| ybtE-R | TGGGAAGACAGCGACCTTTC |
| ybtT-F | GTGCCGCCAGTTGTGTAATG |
| ybtT-R | GCTGAGCAACTGGCTGATTG |
| ybtS-F | GCGCTCGTTTATGTTCCGTC |
| ybtS-R | CGCCTTATTATGCTCCGGGT |
| ybtP-F | GAGTGGGTCGATGTGCTCAA |
| ybtP-R | CATTGGCATTACGGCTTCGG |
| ybtQ-F | CGGGTGAAATGAACGCCTTGC |
| ybtQ-R | AGCCGCCAGTCTATCCACAATG |
| ybtA-F | GCAGTTTCTGGCGTTCCATC |
| ybtA-R | AACCCACTTAACGGCTCAGG |
| 16S rRNA-F | ATTAGATACCCTGGTAGTCCACGC |
| 16S rRNA-R | TTGCGGGACTTAACCCAAC |
| check-cas-F | TCCGAGTCATCCATGCAAAAC |
| check-cas-R | TAACGCAAGTGGAGTAGATGC |
| RE-sg-F | GTGATGACGGTGAAAACCTCTG |
| RE-sg-R | CAGAGGTTTTCACCGTCATCAC |
| irp2-sg-F | CTCTCTTCATGTAACTGCTGGTTTTAGAGCTAGAAATAGCAAG |
| irp2-sg-R | CAGCAGTTACATGAAGAGAGACTAGTATTATACCTAGGACTGAG |
| NDM5-sg-F | AGCGCAGCTTGTCGGCCATGGTTTTAGAGCTAGAAATAGCAAG |
| NDM5-sg-R | CATGGCCGACAAGCTGCGCTACTAGTATTATACCTAGGACTGAG |
| check-sg-R | CTTTACACTTTATGCTTCCGGC |
| irp2-F1 | GGCGAAAACGCCGGTCTTAT |
| irp2-R1 | CGCGAAGCAAACTGATTTTCC |
| irp2-F2 | TTCCGGGGAAAATCAGTTTGCTTCGCGGTTTATTTATGCCCGTTTCGGGT |
| irp2-R2 | TGCTGGATGAACATCGCGAAG |
| check-irp2-F | GGTAGGTACTCATCCGGGAAGA |
| check-irp2-R | TCTGGTCATGCTCAGCCTGG |
| check-NDM5-F | ATGGCTCATCACGATCATGCTG |
| check-NDM5-R | GACGATTGGCCAGCAAATGG |
| pET28-F | CAAAGCCCGAAAGGAAGCTGAG |
| pET28-R | CTGGTGGACAGCAAATGGGT |
| NDM5-ET28-F | CTGGTGGACAGCAAATGGGTATGGAATTGCCCAATATTATGCACCCGGT |
| NDM5-ET28-R | CAGCTTCCTTTCGGGCTTTGTCAGTGATGATGATGATGATGGCGCAGCTTGTCGGCCAT |
| check-pET28-F | GAAGGAGATATACCATGGGCAGC |
| check-pET28-R | GCCAATCCGGATATAGTTCCTCC |

**Supplementary Data 1. (separate file)**

Metadata of sample details and genetic characteristics of ST410 isolates. Each row corresponds to a unique isolate, with metadata including isolate ID, subclone designation, year of collection, geographical location, isolation source, *fimH* allele, O-antigen type, QRDR mutation site, antimicrobial resistance genes, virulence determinants, and plasmid replicons.

**Supplementary Data 2. (separate file)**

Core genome SNP analysis of ST410 isolates, with the ST410 genome (CP104851.1) as the reference. Sheet 1: Hierarchical Bayesian clustering of ST410 isolates. Sheet 2: ANI values (%) of ST410 isolates. Sheet 3: Pairwise SNP distances of ST410 isolates.

**Supplementary Data 3. (separate file)**

HPI prevalence and *bla*_NDM_ prevalence in *E. coli* isolates from the GenBank database. Sheet 1: HPI prevalence. Each row represents a unique isolate, containing data on isolate ID and sequence types. Sheet 2: *bla*_NDM_ prevalence in HPI-positive and HPI-negative strains. Each row represents a unique isolate, containing data on isolate ID, sequence types, and detected *bla*_NDM_ alleles.

**Supplementary Data 4. (separate file)**

Raw data and statistical results for figures in the manuscript, including Figure 1, Figure 2, Figure 5, and Figure 6.

**References**

1.	He WY, Lv LC, Pu WX *et al.* Characterization of an international high-risk *Escherichia coli* ST410 clone coproducing NDM-5 and OXA-181 in a food market in China. *Microbiol Spectr* 2023;**11**:e0472722. https://doi.org/10.1128/spectrum.04727-22

2.	He W, Gao M, Lv L *et al.* Persistence and molecular epidemiology of *bla*_NDM_-positive Gram-negative bacteria in three broiler farms: A longitudinal study (2015–2021). *J Hazard Mater* 2023;**446**:130725. https://doi.org/10.1016/j.jhazmat.2023.130725

3.	Wang Y, Wang S, Chen W *et al.* CRISPR-Cas9 and CRISPR-assisted cytidine deaminase enable precise and efficient genome editing in *Klebsiella pneumoniae*. *Appl Environ Microbiol* 2018;**84**:e01834-18. https://doi.org/10.1128/AEM.01834-18
